# Phase-separated nucleocapsid protein of SARS-CoV-2 suppresses cGAS-DNA recognition by disrupting cGAS-G3BP1 complex

**DOI:** 10.1038/s41392-023-01420-9

**Published:** 2023-04-26

**Authors:** Sihui Cai, Chenqiu Zhang, Zhen Zhuang, Shengnan Zhang, Ling Ma, Shuai Yang, Tao Zhou, Zheyu Wang, Weihong Xie, Shouheng Jin, Jincun Zhao, Xiangdong Guan, Jianfeng Wu, Jun Cui, Yaoxing Wu

**Affiliations:** 1 Guangdong Province Key Laboratory of Pharmaceutical Functional Genes, The First Affiliated Hospital of Sun Yat-sen University, School of Life Sciences, Sun Yat-sen University, Guangzhou, Guangdong China; 2 MOE Key Laboratory of Gene Function and Regulation, State Key Laboratory of Biocontrol, School of Life Sciences, Sun Yat-sen University, Guangzhou, Guangdong China; 3 State Key Laboratory of Respiratory Disease, Guangzhou Institute of Respiratory Disease, The First Affiliated Hospital of Guangzhou Medical University, Guangzhou, Guangdong China; 4 Department of Critical Care Medicine, The First Affiliated Hospital of Sun Yat-sen University, Guangzhou, Guangdong China

**Keywords:** Infectious diseases, Cell biology, Microbiology

## Abstract

Currently, the incidence and fatality rate of SARS-CoV-2 remain continually high worldwide. COVID-19 patients infected with SARS-CoV-2 exhibited decreased type I interferon (IFN-I) signal, along with limited activation of antiviral immune responses as well as enhanced viral infectivity. Dramatic progresses have been made in revealing the multiple strategies employed by SARS-CoV-2 in impairing canonical RNA sensing pathways. However, it remains to be determined about the SARS-CoV-2 antagonism of cGAS-mediated activation of IFN responses during infection. In the current study, we figure out that SARS-CoV-2 infection leads to the accumulation of released mitochondria DNA (mtDNA), which in turn triggers cGAS to activate IFN-I signaling. As countermeasures, SARS-CoV-2 nucleocapsid (N) protein restricts the DNA recognition capacity of cGAS to impair cGAS-induced IFN-I signaling. Mechanically, N protein disrupts the assembly of cGAS with its co-factor G3BP1 by undergoing DNA-induced liquid-liquid phase separation (LLPS), subsequently impairs the double-strand DNA (dsDNA) detection ability of cGAS. Taken together, our findings unravel a novel antagonistic strategy by which SARS-CoV-2 reduces DNA-triggered IFN-I pathway through interfering with cGAS-DNA phase separation.

## Introduction

Currently, the outbreak of coronavirus disease 2019 (COVID-19), which caused by severe acute respiratory syndrome coronavirus 2 (SARS-CoV-2), has been declared a worldwide pandemic.^1–3^ As of 5th January 2023, The COVID-19 pandemic has led to over 600 million confirmed cases and more than 7 million deaths globally. Patients with moderate and severe cases could be distinguished by clinical symptoms. Moderate cases of COVID-19 showed dysfunctional inflammatory responses in lungs, while severe cases always resulted in adult respiratory distress syndrome (ARDS), cytokine storm and even death.^4,5^ Although vaccines were developed with different strategies and showed over 50% protection than the inactive comparator, different variants of SARS-CoV-2 were still spreading and wreaking havoc on human health, which resulted in global medical pressure, implying an urgent threat of public health.^6–8^ Therefore, expansion of the understanding on host–pathogen biology of SARS-CoV-2 for novel and effective therapeutic interventions is still a pressing and urgent task.

Innate immunity applies pattern recognition receptors (PRRs) to detect pathogen associated molecular patterns (PAMPs) and damage associated molecular patterns (DAMPs) to activate the effective immune responses and downstream antiviral responses against viruses.^9^ Among PRRs, cGAS, the cyclic GMP-AMP (cGAMP) synthase, recognizes cytosolic dsDNA from cellular/mitochondrial origin or dsDNA that introduced by invading pathogen particles, such as bacteria and viruses.^10,11^ Once detecting and binding with cytosolic DNA, cGAS catalyzes the synthesis of cGAMP to induce the translocation of stimulator of interferon genes (STING), thereby promoting the phosphorylation of tank-binding kinase 1 (TBK1). The phosphorylated TBK1 then mediates the phosphorylation of interferon regulatory factor 3 (IRF3), leading to the transcription of interferon β (IFNβ) and subsequently inducing the expression of interferon-stimulated genes (ISGs).^12^ In addition to initiating type I interferon (IFN-I) pathway against DNA viruses, cGAS could also sense reverse-transcribed DNA or released mitochondria DNA (mtDNA) during Retrovirus, Dengue virus (DENV) or Zika virus (ZIKV) infection.^13–16^ Recently, researches have broadened the acquaintance on the involvement of cGAS-mediated signaling pathway during infection and replication of SARS-CoV-2. Mitochondria damage or nuclei damage caused by SARS-CoV-2 triggered mtDNA release or chromatin DNA shuttled from the nucleus, which led to the accumulation of cytosolic dsDNA and cGAS-mediated IFN-I signaling activation, inflammatory responses, or STING-dependent cell death.^17–21^ However, the antagonistic strategies of SARS-CoV-2 against cGAS signaling pathway during infection remain unknown.

As a coronaviridae family member, SARS-CoV-2 possesses a genome encoding 14 major open reading frames (ORFs). These ORFs can be processed into 16 nonstructural proteins (NSP1 to 16), 4 structural proteins, including nucleocapsid (N) protein, spike protein, envelope protein, membrane protein and 9 accessory proteins.^22^ N protein is the most abundant viral protein and the core element of virions during infection, which serves as a promising drug design target.^23^ Recent studies suggest that N protein from SARS-CoV-2 could enter and disrupt G3BP stress granule assembly factor 1 (G3BP1)-containing condensates, the cofactor of cGAS.^24–26^ It provokes our attention to the antagonisms between SARS-CoV-2 infection and cGAS-triggered IFN-I signaling.

In the current study, we uncover the engagement of cGAS-mediated signaling in SARS-CoV-2-induced IFN-I by detecting cytosolic mtDNA released by damaged mitochondria. To inhibit cGAS-mediated antiviral signaling, N protein suppresses the DNA recognition ability of cGAS during SARS-CoV-2 infection. Mechanically, DNA-induced liquid-liquid phase separation (LLPS) of N protein competitively binds with G3BP1, a co-factor of cGAS, resulting in the disruption of cGAS-G3BP1 complex and the impairment of cGAS-DNA detection. We unveil a novel strategy employed by SARS-CoV-2 in antagonizing DNA-induced innate immune signaling pathway, providing a possible therapeutic target for treatment against SARS-CoV-2 infection.

## Results

### SARS-CoV-2 infection induces mtDNA release and recognition by cGAS

**Fig. 1:**
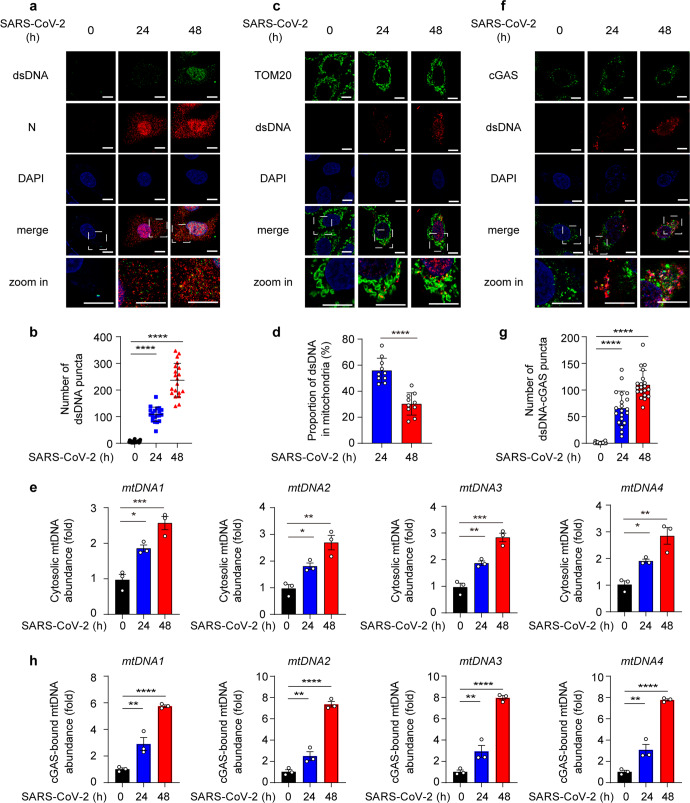
SARS-CoV-2 infection induces mtDNA release and cGAS-dsDNA detection. **a**, **b** Representative confocal micrographs of ACE2-expressing A549 cells infected with SARS-CoV-2 (MOI = 0.1) for indicated time points, followed by labeling double-strand DNA (dsDNA, green) and SARS-CoV-2 N protein (red) (**a**). Scale bars indicated 10 μm. The number of dsDNA puncta per cell (**b**) were counted and analyzed from *n* = 20 cells. **c**, **d** Representative confocal micrographs of ACE2-expressing A549 cells infected with SARS-CoV-2 (MOI = 0.1) for indicated time points, followed by labeling mitochondria (TOM20, green) and double-strand DNA (dsDNA, red) (**c**). Scale bars indicated 10 μm. The proportion of dsDNA in mitochondria (**d**) were counted and analyzed from *n* = 10 cells. **e** Quantitative real-time PCR (qRT-PCR) of cytosolic mtDNA abundance of Calu3 cells infected with SARS-CoV-2 (MOI = 0.1) for indicated time points. **f**, **g** Representative confocal micrographs of ACE2-expressing A549 cells infected with SARS-CoV-2 (MOI = 0.1) for indicated time points, followed by labeling cGAS (green) and double-strand DNA (dsDNA, red) (**f**). Scale bars indicated 10 μm. The number of dsDNA-cGAS puncta per cell (**g**) were counted and analyzed from *n* = 20 cells. **h** Calu3 cells were infected with SARS-CoV-2 (MOI = 0.1) for indicated time points. Cell lysates were collected, immunoprecipitated with A + G beads together with cGAS antibody, followed by qRT-PCR analysis of extracted DNA to detect cGAS-bound mtDNA abundance. Data in **b**, **g** were expressed as mean ± SD of 20 cells for each condition. Data in **d** were expressed as mean ± SD of 10 cells for each condition. Data in **e**, **h** were expressed as mean ± SEM of 3 independent biological experiments. *P < 0.05, **P < 0.01, ***P < 0.001, ****P < 0.0001, ns not significant (unpaired two-tailed student’s *t*-test). Similar results were obtained for 3 independent biological experiments in (**a**, **c**, **f**)

Recent studies revealed that SARS-CoV-2 stimulation could cause the accumulation of intracellular and intercellular DNA due to cell fusion, cell death and even tissue damage.^17,19,27^ To detect SARS-CoV-2-triggered release of DNA during infection, we first detected the cytosolic dsDNA pattern by fluorescent microscopy. In ACE2-expressing A549 cells and Huh7 cells, we found the density and intensity of dsDNA foci in the cytoplasm were dramatically augmented at 24 h and 48 h after SARS-CoV-2 infection (Fig. 1a, b, Supplementary Fig. 1a, b). SARS-CoV-2 infection has been shown to promote chromatin nucleus-to-cytoplasm trafficking and micronuclei formation due to syncytium formation.^17,19–21,27^ To check whether DNA release from syncytia, we detected the syncytium formation in ACE2-expressing A549 cells and Calu3 cells. Our results showed that only a few syncytia were discovered after SARS-CoV-2 infection, suggesting that syncytia formation might not the main source of accumulated cytosol dsDNA in these cells (Supplementary Fig. 1c, d). SARS-CoV-2 infection have been proven to accompany with mitochondria damage,^19,27–29^ which might result in the mtDNA release. To verify SARS-CoV-2 triggered mitochondria dysfunction and mitochondria-mediated apoptosis, we used SARS-CoV-2 to stimulate Calu3 and checked the apoptosis marker using quantitative real-time PCR (qRT-PCR) and immunoblotting. We found the cleavages of caspase-3 and PARP were promoted (Supplementary Fig. 1e). Consistently, we found the decreased mRNA level of *BCL-2*, the anti-apoptotic protein, together with the enhanced mRNA level of pro-apoptotic proteins, such as BAX and BAK, which localized on the mitochondria outer membrane and regulated the permeability of mitochondria membrane as treated with SARS-CoV-2 (Supplementary Fig. 1f). We also detected the cytosolic dsDNA pattern along with TOM20, the mitochondrial membrane protein, using fluorescent microscopy after SARS-CoV-2 stimulation, and found that mitochondria identified with TOM20 were ruptured and broken by the SARS-CoV-2, while the density of dsDNA was increased and escaped from mitochondria, indicating the mtDNA release from mitochondria to cytosol due to mitochondria damage (Fig. 1c, d). The increasing level of mtDNA abundance was further verified in Calu3 cells infected with SARS-CoV-2 (Fig. 1e). Our data suggested that SARS-CoV-2 stimulation led to the accumulation of mtDNA in cytosol.

Given that cGAS serves as a pivotal DNA sensor by detecting cytosolic DNA to activate innate immune signaling and antiviral response, including IFN-I signaling pathway, we wondered whether accumulated cytosolic dsDNA could be captured by cGAS. The association between cGAS and cytosolic dsDNA was detected, and the result showed the co-localization between cGAS and cytosolic dsDNA was increased (Fig. 1f, g). To quantify the abundance of cGAS-bound mtDNA, we pulled down cGAS and determined the level of mtDNA by qRT-PCR, and found that the cGAS-bound mtDNA level was enhanced after SARS-CoV-2 stimulation (Fig. 1h). Collectively, SARS-CoV-2 induced mitochondria dysfunction and apoptosis, accompanied with mtDNA release and accumulation at cytoplasm and interaction with DNA sensor cGAS.

### cGAS contributes to SARS-CoV-2-triggered activation of IFN-I responses

As cGAS recognized SARS-CoV-2-triggered mtDNA release, we sought to determine the importance and engagement of the cGAS-mediated IFN-I responses during anti-SARS-CoV-2 innate immunity. RIG-I (encoded by *DDX58*) and MDA5 (encoded by *IFIH1*) are the major receptors to sense SARS-CoV-2 RNA and contribute to antiviral responses.^30–32^ Knocking down of *DDX58* or *IFIH1* inhibited the mRNA level of *IFNβ* and downstream cytokines, such as *IFIT1* and *IFIT2*, and dramatically enhanced the virus replication level due to the inhibition of antiviral responses (Supplementary Fig. 2a–d). But we also showed that SARS-CoV-2 still triggered a certain level of *IFNβ* and ISGs expression in RIG-I like receptors (RLRs)-deficient cells, implying an RLRs-independent activation of IFN-I pathway.

**Fig. 2:**
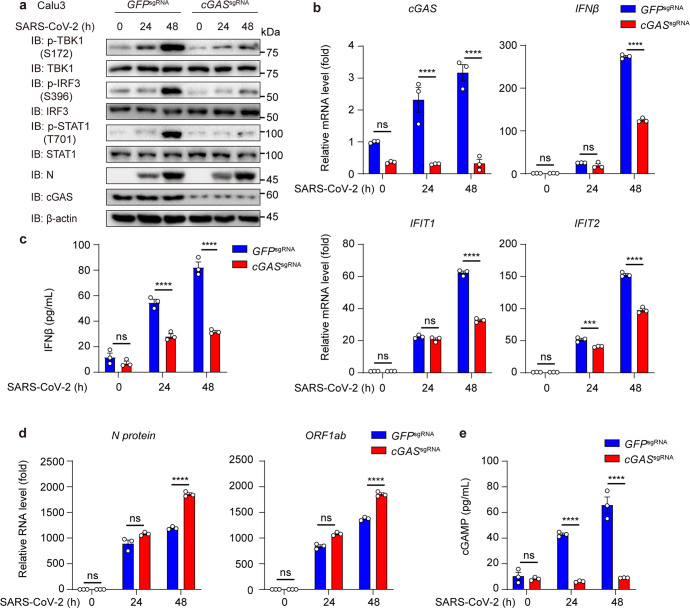
SARS-CoV-2 infection activates cGAS-mediated IFN-I signaling. **a** Immunoblot analysis of *GFP*^sgRNA^ and *cGAS*^sgRNA^ Calu3 cells infected with SARS-CoV-2 (MOI = 0.1) for indicated time points. **b** qRT-PCR with reverse transcription analysis of *cGAS, IFNβ, IFIT1,* and *IFIT2* mRNA level of *GFP*^sgRNA^ and *cGAS*^sgRNA^ Calu3 cells with SARS-CoV-2 (MOI = 0.1) infection for indicated time points. **c** Cellular IFNβ production of *GFP*^sgRNA^ and *cGAS*^sgRNA^ Calu3 cells infected with SARS-CoV-2 (MOI = 0.1) for indicated time points detected by ELISA assay. **d** qRT-PCR with reverse transcription analysis of *N protein* and *ORF1ab* RNA of SARS-CoV-2 in supernatant of *GFP*^sgRNA^ and *cGAS*^sgRNA^ Calu3 cells with SARS-CoV-2 (MOI = 0.1) infection for indicated time points. **e** Cellular cGAMP production of *GFP*^sgRNA^ and *cGAS*^sgRNA^ Calu3 cells infected with SARS-CoV-2 (MOI = 0.1) for indicated time points detected by ELISA assay. Data in **b**–**e** were expressed as mean ± SEM of 3 independent biological experiments. ***P < 0.001, ****P < 0.0001, ns not significant (unpaired two-tailed student’s *t*-test). Similar results were obtained for 3 independent biological experiments in **a**

To determine the participation of cGAS, we generated *cGAS*^*s*gRNA^ Calu3 cells to investigate whether *cGAS* deficiency affected SARS-CoV-2-stimulated activation of IFN-I responses. Deficiency of *cGAS* led to the reduction of phosphorylation level of TBK1, IRF3, and STAT1, the mRNA level of *IFNβ* and ISGs, and the cellular IFNβ productions, suggesting that cGAS-dependent pathway contributed on SARS-CoV-2-stimulated activation of IFN responses (Fig. 2a–c and S2e). In addition, the increased SARS-CoV-2 *N protein* and *ORF1ab* RNA level were observed by qRT-PCR assay in *cGAS* deficiency cells (Fig. 2d). Moreover, consistent with previous reports,^17^ increased cGAMP production levels were found in Calu3 cells after stimulation of SARS-CoV-2, while the production of cGAMP was dramatically inhibited in *cGAS*-deficient cells (Fig. 2e). Collectively, our findings revealed that DNA sensor cGAS participated in activation of IFN-I responses to restrict virus replication during SARS-CoV-2 invasion.

### N protein counteracts the cGAS-induced IFN-I pathway by impairing cGAS-dsDNA association

**Fig. 3:**
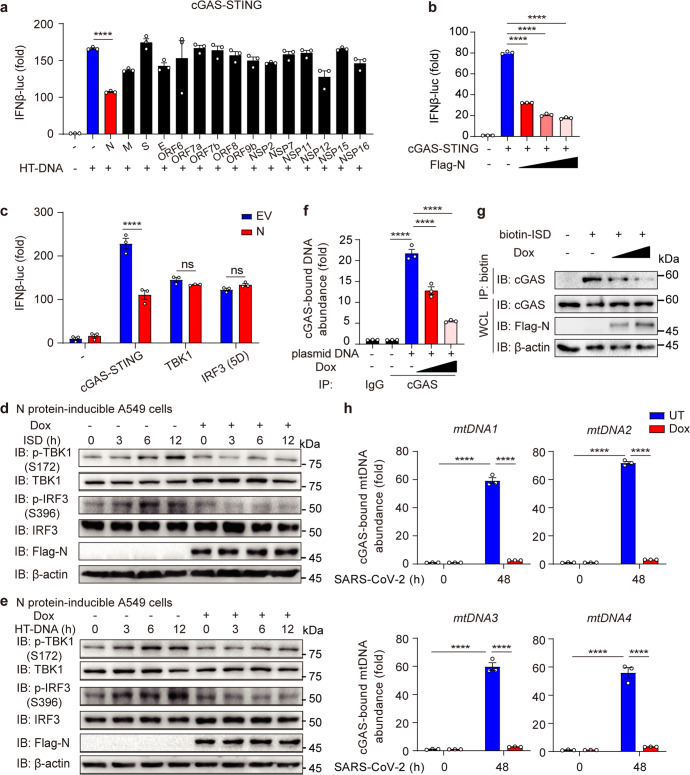
SARS-CoV-2 nucleocapsid protein counteracts cGAS-mediated IFN-I signaling by dampening cGAS-DNA recognition. **a** HEK-293T cells transfected with IFNβ luciferase reporter (IFNβ-luc) and TK-luc, Flag-GFP-cGAS and HA-STING, along with plasmids expressing SARS-CoV-2 viral protein were treated with HT-DNA for 12 h. Cell lysates were collected and luciferase assay was performed. **b** HEK-293T cells were transfected with IFNβ-luc, TK-luc, Flag-GFP-cGAS and HA-STING, along with increasing expressed SARS-CoV-2 N protein. Cell lysates were collected and luciferase assay was performed. **c** HEK-293T cells were transfected with IFNβ-luc, TK-luc, plasmids expressing cGAS, STING, TBK1 or IRF3 (5D), along with Flag-empty vector (EV) or Flag-N. Cell lysates were collected and luciferase assay was performed. **d** Immunoblot analysis of N protein-inducible A549 cells treated with doxycycline (Dox, 200 ng/mL) for 24 h or left untreated (UT) with ISD (2 μg/mL) treatment for indicated time points. **e** Immunoblot analysis of N protein-inducible A549 cells treated with doxycycline (Dox, 200 ng/mL) for 24 h or left untreated with HT-DNA (2 μg/mL) treatment for indicated time points. **f** N protein-inducible Calu3 cells treated with increasing concentration of Dox (200 ng/mL and 400 ng/mL) were transfected with mCherry plasmid (2 μg/mL) as plasmid DNA for 2 h before harvest. Cell lysates were collected, immunoprecipitated with A + G beads together with cGAS antibody, followed by qRT-PCR analysis of extracted DNA to detect cGAS-bound plasmid DNA (*mCherry*) abundance. **g** N protein-inducible Calu3 cells treated with increasing concentration of Dox (200 ng/mL and 400 ng/mL) and transfected with biotin-ISD (2 μg/mL) stimulation for 2 h before harvest. Cell lysates were collected, immunoprecipitated with NeutrAvidin agarose resin, followed by immunoblotting analysis. **h** N protein-inducible Calu3 cells treated with Dox (200 ng/mL) or left UT were infected by SARS-CoV-2 (MOI = 0.1) for indicated time points. Cell lysates were collected, immunoprecipitated with A + G beads together with cGAS antibody, followed by qRT-PCR analysis of extracted DNA to detect cGAS-bound mtDNA abundance. Data in **a**–**c**, **f**, **h**) were expressed as mean ± SEM of 3 independent biological experiments. ****P < 0.0001, ns not significant (unpaired two-tailed student’s *t*-test). Similar results were obtained for 3 independent biological experiments in **d**, **e**, **g**

Since we and other researchers uncovered that the cGAS-dependent signaling exhibited a vital role in IFN-I induction and viral restriction against invading SARS-CoV-2,^17,19^ we wondered whether SARS-CoV-2 had evolved any tactics to inhibit the cGAS-mediated pathway. We tried to detect different effects of SARS-CoV-2 viral proteins in restricting cGAS-triggered IFN induction. We found N protein significantly inhibited the IFNβ luciferase (IFNβ-luc) activity induced by HT-DNA, which specifically triggered the cGAS-mediated pathway (Fig. 3a). The reduction of cGAS-STING-induced IFNβ-luc level was detected with increasing expression level of N protein (Fig. 3b), suggesting that N protein functions in inhibiting cGAS-STING pathway.

N protein was previously reported to suppress the MAVS-dependent IFN-I signaling pathway,^33^ but the involvement and regulation of N protein in cGAS-STING signaling pathway remained unclear. We transfected plasmids encoding N protein together with the established signaling molecules which function sequentially in cGAS-mediated pathway, such as cGAS/STING, TBK1, or IRF3 (5D), and showed N protein inhibited IFNβ-luc at cGAS and STING level (Fig. 3c). To further depict the functions of N protein, we generated N protein-inducible A549 cell line and investigated whether N protein could affect the cGAS-induced IFN-I signaling after stimulation of interferon stimulatory DNA (ISD), a 45 bp dsDNA that directly triggered cGAS. Our results showed that N protein reduced the phosphorylation levels of TBK1 and IRF3 after ISD treatment (Fig. 3d and Supplementary Fig. 3a). Similarly, N protein also restricted the phosphorylation levels of TBK1 and IRF3 after HT-DNA stimulation (Fig. 3e and Supplementary Fig. 3b). These findings together demonstrated that N protein of SARS-CoV-2 counteracted cGAS-triggered activation of IFN-I responses.

In order to figure out how N protein suppresses cGAS-STING pathway, we checked whether N protein affected the protein level of cGAS. Unexpectedly, ectopic N protein didn’t significantly influence the protein level of cGAS (Supplementary Fig. 3c). We subsequently generated N protein-inducible Calu3 cell line and investigated whether N protein dampened IFN responses by affecting cGAS-DNA interaction. By pulling down cGAS and testing cGAS-bound plasmid DNA level through qRT-PCR, we found that N protein hindered the DNA binding capacity of cGAS (Fig. 3f). Consistently, by capturing biotin-ISD binding proteins with NeutrAvidin agarose resin, we observed that ectopic N protein also significantly reduced the biotin-ISD-bound cGAS level (Fig. 3g). Compared to other viral structural components, N protein dramatically disrupted cGAS-bounded dsDNA abundance, while S, E and M proteins slightly affected the interaction between cGAS and dsDNA (Supplementary Fig. 3d). We next infected N protein-inducible Calu3 cells with SARS-CoV-2 and detected cGAS-bound mtDNA abundance, and found that N protein modestly increased the virus-induced mtDNA release in the cytosol, but dramatically reduced the cGAS-bound mtDNA level (Fig. 3h and Supplementary Fig. 3e). Consistently, fluorescent microscopy analysis revealed that cGAS-DNA puncta were reduced as the accumulation of N protein under HT-DNA stimulation (Supplementary Fig. 3f). Altogether, these findings revealed that N protein hampered the DNA recognition of cGAS and thus inhibiting cGAS-induced IFN-I signaling.

### N protein restricts cGAS functions through G3BP1

Since N protein inhibits cGAS-DNA interaction and IFN-I responses in cells, we sought to determine whether N protein directly influences cGAMP production. We generated and purified recombinant cGAS protein and N protein by *E. coli* system and conducted in vitro experiment to detect cGAMP processing ability by testing ATP consumption. Surprisingly, while the mixture of cGAS and DNA induced the cGAMP processing ability of cGAS, the addition of N protein could not regulate this process in the cell-free system (Supplementary Fig. 4a), suggesting that N protein was not directly regulated cGAS function.

**Fig. 4:**
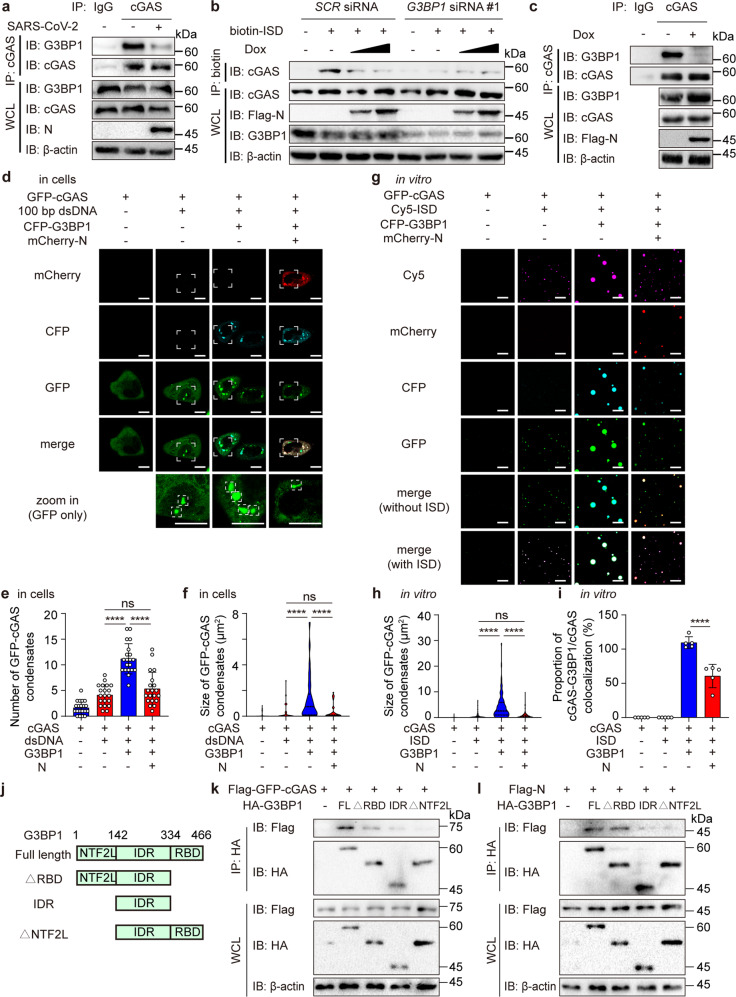
SARS-CoV-2 nucleocapsid protein suppresses cGAS-G3BP1 interaction. **a** Calu3 cells were infected with SARS-CoV-2 (MOI = 0.1) for 0 or 24 h. Cell lysates were collected, immunoprecipitated with A + G beads along with cGAS antibody, followed by immunoblotting analysis. **b** N protein-inducible Calu3 cells transfected with *scramble* (*SCR*) siRNA or *G3BP1* siRNA #1, were treated with increasing concentration of Dox (200 ng/mL and 400 ng/mL) for 24 h with biotin-ISD treatment for 2 h before harvest. Cell lysates were collected, immunoprecipitated with NeutrAvidin agarose resin, followed by immunoblotting analysis. **c** N protein-inducible Calu3 cells were treated with Dox (200 ng/mL) or left untreated. Cell lysates were collected, immunoprecipitated with A + G beads together with cGAS antibody, followed by immunoblotting analysis. **d**–**f** Representative images of confocal microscopy in HeLa cells expressing GFP-cGAS, mCherry-N and CFP-G3BP1 with 100 bp dsDNA stimulation (2 μg/mL) for 12 h before harvest (**d**). Scale bars indicated 10 μm. The number of GFP-cGAS condensates (**e**) were analyzed from *n* = 20 cells and the size of GFP-cGAS condensates per cell (**f**) were measured from *n* = 30 condensates. **g**–**i** Representative images of confocal microscopy of recombinant GFP-cGAS (10 μM, green), CFP-G3BP1 (10 μM, cyan), mCherry-N (5 μM, red) and Cy5-ISD (2 μM, magenta) incubated in LLPS buffer at 37 °C (**g**). Scale bars indicated 10 μm. The size of GFP-cGAS condensates (**h**) was measured from *n* = 200 condensates and the proportion of cGAS-G3BP1 co-localized condensates/cGAS condensates (**i**) were analyzed from *n* = 5 views. **j** Schematic figure of domain organization of G3BP1 and its domain deletion mutant. **k** HEK-293T cells were transfected with Flag-empty vector (EV) or Flag-GFP-cGAS along with HA-EV, HA-G3BP1 full length (FL), △RBD, IDR and △NTF2L. Cell lysates were collected, immunoprecipitated with Flag-beads, followed with immunoblotting analysis. **l** HEK-293T cells were transfected with Flag- EV or Flag-N along with HA-EV, HA-G3BP1 FL, △RBD, IDR and △NTF2L. Cell lysates were collected, immunoprecipitated with Flag-beads, followed with immunoblotting analysis. Data in **e**, **i** were expressed as mean ± SD of indicated samples for each condition. Data in **f**, **h** were expressed as median and quartiles of indicated samples for each condition. ****P < 0.0001, ns not significant (unpaired two-tailed student’s *t*-test). Similar results were obtained for 3 independent biological experiments in **a**–**d**, **g**, **k**, **l**

We next sought to investigate how N protein restricted DNA binding ability of cGAS. G3BP1, a well-established co-factor of cGAS, which recently proved to exhibit a strong interaction with SARS-CoV-2 N protein, caught our attention.^24,25^ G3BP1 was shown to be associated with cGAS to promote the recognition between cGAS and DNA, thereby increasing the IFN-I signaling level.^34–36^ We found SARS-CoV-2 stimulation promoted the disassembly of cGAS and its co-factor G3BP1 (Fig. 4a). To identify the involvement of G3BP1 during SARS-CoV-2 invasion, we examined IFN-I activation after SARS-CoV-2 stimulation in *G3BP1*-knockdown Calu3 cells, and found that deficiency of *G3BP1* led to reduced phosphorylation levels of TBK1, IRF3 and STAT1 under SARS-CoV-2 stimulation (Supplementary Fig. 4b, c). Consistently, SARS-CoV-2 induced expression levels of *IFNβ*, *ISG15*, *IFIT1* and *IFIT2* were reduced in *G3BP1*-knockdown Calu3 cells (Supplementary Fig. 4d), suggesting the engagement of G3BP1 in SARS-CoV-2-induced activation of IFN-I responses.

Since the association between cGAS and G3BP1 was important for cGAS-DNA recognition,^34,35^ we next examined whether N protein restricted cGAS-DNA interaction through G3BP1. Although N protein remarkably attenuated the interaction between cGAS and ISD in controlled cells, N protein failed to affect cGAS-ISD association in *G3BP1*-deficient cells (Fig. 4b and S4e). By in vitro experiment, we also found that G3BP1 significantly provoked the cGAMP processing ability of cGAS, while N protein inhibited cGAS ability in the presence of G3BP1 (Supplementary Fig. 4f). We next found that N protein dramatically hindered the association between cGAS and G3BP1 (Fig. 4c). Fluorescence experiments showed that G3BP1 magnified cGAS-DNA recognition ability by promoting the number and the size of cGAS-DNA condensates, while N protein formed condensates and competitively interacted with G3BP1 to exclude cGAS from N protein-G3BP1 condensates and restrict the number and the size of cGAS-DNA LLPS condensates in cells (Fig. 4d–f). We next performed in vitro experiments by mixing the recombinant GFP-cGAS, CFP-G3BP1 and mCherry-N protein, along with Cy5-ISD. Consistently, we found N protein also formed the condensates, clustered and co-localized with G3BP1. By analyzing the proportion of cGAS-G3BP1/cGAS condensates and the size of cGAS condensates, we discovered that N protein reduced the co-localization between cGAS and G3BP1, consequently limiting the size of cGAS-DNA LLPS condensates in vitro (Fig. 4g–i).

To explore the mechanisms that N protein utilized to restrict cGAS pathway via G3BP1, we generated plasmids expressing domain deletion mutants of G3BP1, including deletion of nuclear transport factor 2-like (NTF2L) domain, deletion of RNA-binding domain (RBD) and intrinsically disordered region (IDR) domain only according to previous studies (Fig. 4j).^26,37^ We discovered that cGAS mainly interacted with the RBD and NTF2L domain of G3BP1 (Fig. 4k). Intriguingly, our immunoprecipitation experiments revealed that N protein also interacted with NTF2L domain of G3BP1 (Fig. 4l), which was confirmed by several previous studies,^26,37^ implying that N protein and cGAS might competitively interact with NTF2L domain of G3BP1. Together, these results suggested that N protein of SARS-CoV-2 competed G3BP1 with cGAS to disrupt cGAS-G3BP1 complex and suppressed the DNA recognition ability of cGAS.

### N protein disrupts cGAS-G3BP1 complex through DNA-induced LLPS

**Fig. 5:**
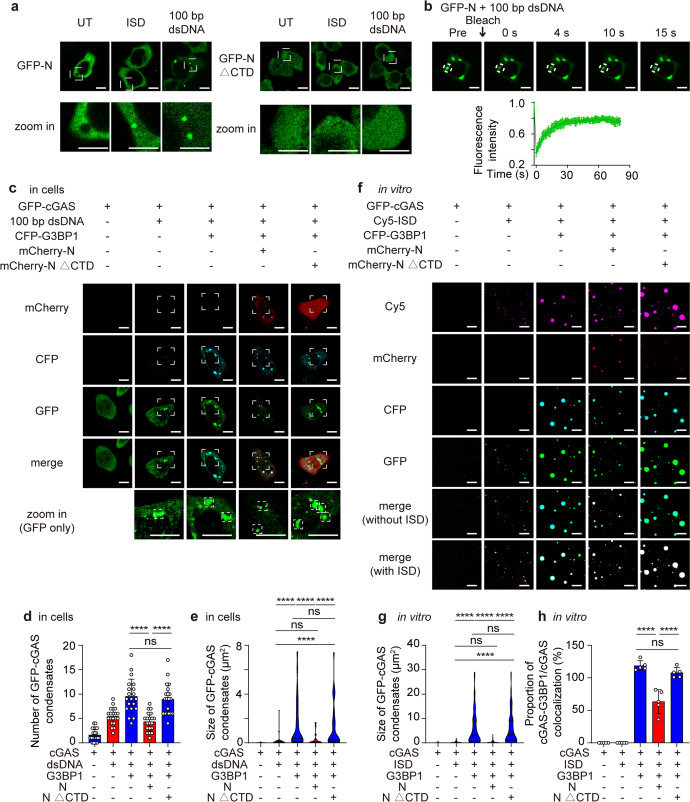
SARS-CoV-2 nucleocapsid protein undergoes DNA-induced LLPS to interfere the formation of cGAS-G3BP1 complex. **a** Representative images of confocal microscopy of HeLa cells expressing GFP-N protein or GFP-N protein △CTD treated with 45 bp ISD (2 μg/mL), 100 bp dsDNA (2 μg/mL) treatment for 12 h. Scale bars indicated 10 μm. **b** Live cell microscopy of HeLa cells expressing GFP-N protein or GFP-N protein △CTD stimulated with 100 bp dsDNA (2 μg/mL) for 12 h. Bleaching was performed at the indicated time points and the recovery occurred at 37 °C. Scale bars indicated 10 μm. Fluorescence intensity analysis of fluorescence recovery after photobleaching (FRAP) over a 90 s time course from *n* = 7 droplets. **c**–**e** Representative images of confocal microscopy in HeLa cells expressing GFP-cGAS, CFP-G3BP1 and mCherry-N or mCherry-N △CTD with 100 bp dsDNA stimulation (2 μg/mL) for 12 h before harvest (**c**). Scale bars indicated 10 μm. The number of GFP-cGAS condensates (**d**) were analyzed from *n* = 20 cells and the size of GFP-cGAS condensates per cell (**e**) were measured from *n* = 30 condensates. **f**–**h** Representative images of confocal microscopy of recombinant GFP-cGAS (10 μM, green), CFP-G3BP1 (10 μM, cyan), mCherry-N (5 μM, red) or mCherry-N △CTD (5 μM, red) along with Cy5-ISD (2 μM, magenta) incubated in LLPS buffer at 37 °C (**f**). Scale bars indicated 10 μm. The size of GFP-cGAS condensates (**g**) was measured from *n* = 200 condensates and the proportion of cGAS-G3BP1 co-localized condensates/cGAS condensates (**h**) were analyzed from *n* = 5 views. Data in **d**, **h** were expressed as mean ± SD of indicated samples for each condition. Data in **e**, **g** were expressed as median and quartiles of indicated samples for each condition. ****P < 0.0001, ns not significant (unpaired two-tailed student’s *t*-test). Similar results were obtained for 3 independent biological experiments in (**a**–**c** and **f**)

N protein of SARS-CoV-2 was reported to bind with RNA and undergo RNA-induced LLPS during viral invasion.^38,39^ In Fig. 4d, g, we observed the N protein-contained liquid phase condensations both in cells and in vitro, which made us wonder whether N protein could undergo LLPS under the stimulation of DNA. To investigate the DNA-induced LLPS ability of N protein and the function of critical domain for N protein-DNA condensation, we performed the LLPS experiments of N protein full length (FL) along with different N protein domain deletion mutants, including the N-terminal domain deletion mutant (△NTD), the central serine- and arginine-rich tract deletion mutant (△SR) and the C-terminal domain deletion mutant (△CTD) as previously described (Supplementary Fig. 5a),^40^ with dsDNA of different lengths in vitro and in cells. Both 45 bp ISD and 100 bp dsDNA could largely induce the formation of mCherry-N protein condensates in cells and in vitro (Fig. 5a and S5b). In vitro experiments also identified that △NTD and △SR mutant of N protein formed condensates with DNA stimulation (Supplementary Fig. 5b), while N protein △CTD mutant only showed a dispersed pattern both in cells and in vitro compared with FL N protein and other N protein mutants (Fig. 5a and S5b). By fluorescence recovery after photobleaching (FRAP) experiments, we found that DNA-induced N protein condensates were liquid-like droplets with decent mobility (Fig. 5b and Supplementary Fig. 5c), suggesting the DNA-induced LLPS property of N protein and the significant effect of CTD domain in N protein condensates formation.

Subsequently, we sought to figure out the relationship between DNA-induced LLPS of N protein and its function of suppressing cGAS-G3BP1 interaction and cGAS-DNA condensates formation. By fluorescence experiments, we found G3BP1 formed the condensates with FL N protein, but not the N protein △CTD mutant (Supplementary Fig. 5d). Subsequently, we compared the function of FL N protein and N protein △CTD mutant by further experiments. Consistent with the results of Fig. 4d–i, FL N protein disrupted cGAS-G3BP1 condensation by interacting and clustering with G3BP1. However, N protein △CTD mutant, which lost its LLPS ability, no longer formed condensates with G3BP1 or restricted the number or size of cGAS-DNA condensates in cells (Fig. 5c–e). In vitro experiments confirmed that LLPS ability-abolished N protein △CTD mutant could not form LLPS condensates, and the analysis of the size of cGAS condensates and the proportion of cGAS-G3BP1/cGAS condensates implied that △CTD mutant could not competitively bind with G3BP1 to hinder the interaction of cGAS-DNA (Fig. 5f–h). These results together demonstrated that N protein restricted the cGAS-G3BP1-DNA complex through DNA-induced LLPS ability.

### SARS-CoV-2 N protein suppresses cGAS-DNA recognition through LLPS ability

In order to define the molecular basis and physiological function of phase separated N protein on cGAS-induced IFN-I signaling, immunoprecipitation assay between G3BP1 and different domain of N protein was conducted. Our results showed that G3BP1 strongly interacted with FL N protein, its △NTD and △SR mutant, but not its LLPS ability-abolished △CTD mutant (Supplementary Fig. 6a).

**Fig. 6:**
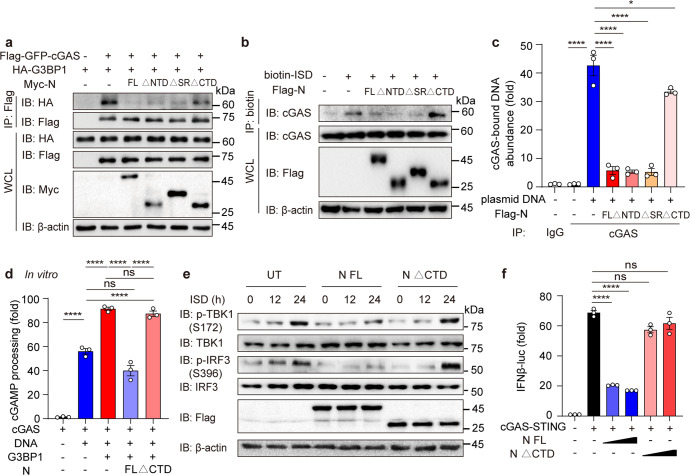
SARS-CoV-2 nucleocapsid protein LLPS is crucial for inhibition of cGAS-mediated IFN-I signaling. **a** HEK-293T cells were expressed with Flag-GFP-cGAS, HA-G3BP1, along with Myc-N-FL, △NTD, △SR or △CTD. Cell lysates were collected, immunoprecipitated with Flag-beads, followed by immunoblotting. **b** Calu3 cells expressed with Flag-N full length (FL), △NTD, △SR or △CTD were stimulated with biotin-ISD (2 μg/mL) for 2 h before harvest. Cell lysates were collected, immunoprecipitated with NeutrAvidin agarose resin, followed by immunoblotting analysis. **c** Calu3 cells expressed with Flag-N FL, △NTD, △SR or △CTD were transfected with mCherry plasmid (2 μg/mL) as plasmid DNA for 2 h before harvest. Cell lysates were collected, immunoprecipitated with A + G beads together with cGAS antibody, followed by qRT-PCR analysis of extracted DNA to detect cGAS-bound plasmid DNA (*mCherry*) abundance. **d** In vitro cGAMP processing ability of recombinant cGAS (10 μM) incubated with recombinant G3BP1 (10 μM), recombinant N protein (5 μM) or recombinant N-△CTD (5 μM) and ISD (2 μM), along with ATP. (**e**) Immunoblotting analysis of N protein-inducible Calu3 cells or N-△CTD-inducible Calu3 cells treated with Dox (200 ng/mL) along with ISD (2 μg/mL) stimulation for indicated time points. **f** HEK-293T cells were transfected with IFNβ-luc, TK-luc along with increasing concentration of FL N protein or N-△CTD. Cell lysates were collected and luciferase analysis was performed. Data in **c**, **d** and **f** were expressed as mean ± SEM of 3 independent biological experiments. *P < 0.05, ****P < 0.0001, ns not significant (unpaired two-tailed student’s *t*-test). Similar results were obtained for 3 independent biological experiments in **a**, **b**, **e**

We further conducted the investigation of cGAS-G3BP1 interaction, and found that FL N protein, △NTD and △SR mutants suppressed the interaction between cGAS and G3BP1 compared to controlled cells and LLPS ability-abolished △CTD mutant (Fig. 6a and S6b), implying that LLPS ability of N protein might be necessary to restrict the association between G3BP1 and cGAS. By pulling-down biotin-ISD, we confirmed that FL N protein, △NTD and △SR mutants blocked the interaction between biotin-ISD and cGAS, while △CTD mutant showed a limited effect on cGAS-ISD binding ability (Fig. 6b and S6c). We also performed the cGAS-bound DNA experiment, and found that cGAS-bound DNA abundance was attenuated with FL N protein, △NTD and △SR mutants, but not △CTD mutant (Fig. 6c), implying the LLPS ability of N protein inhibited cGAS-DNA interaction, which was consistent with the results of cGAS-G3BP1 interaction.

We then wanted to elaborate the physiological function of N protein LLPS. In vitro experiments showed that G3BP1 promoted the cGAMP processing ability of cGAS, while N protein but not its LLPS ability-abolished mutant (△CTD) inhibited this process (Fig. 6d). Compared with FL N protein, its △CTD mutant no longer reduced the phosphorylation levels of TBK1 and IRF3 with ISD stimulation in cells (Fig. 6e and S6d). IFNβ-luc experiments confirmed that FL N protein but not its △CTD mutant reduced IFNβ-luc level (Fig. 6f), suggesting that LLPS ability of N protein was critical to restrict cGAS signaling pathway. Altogether, these results verified that N protein dampened cGAS-triggered IFN-I activation by hindering cGAS-DNA association through DNA-induced LLPS ability.

**Fig. 7:**
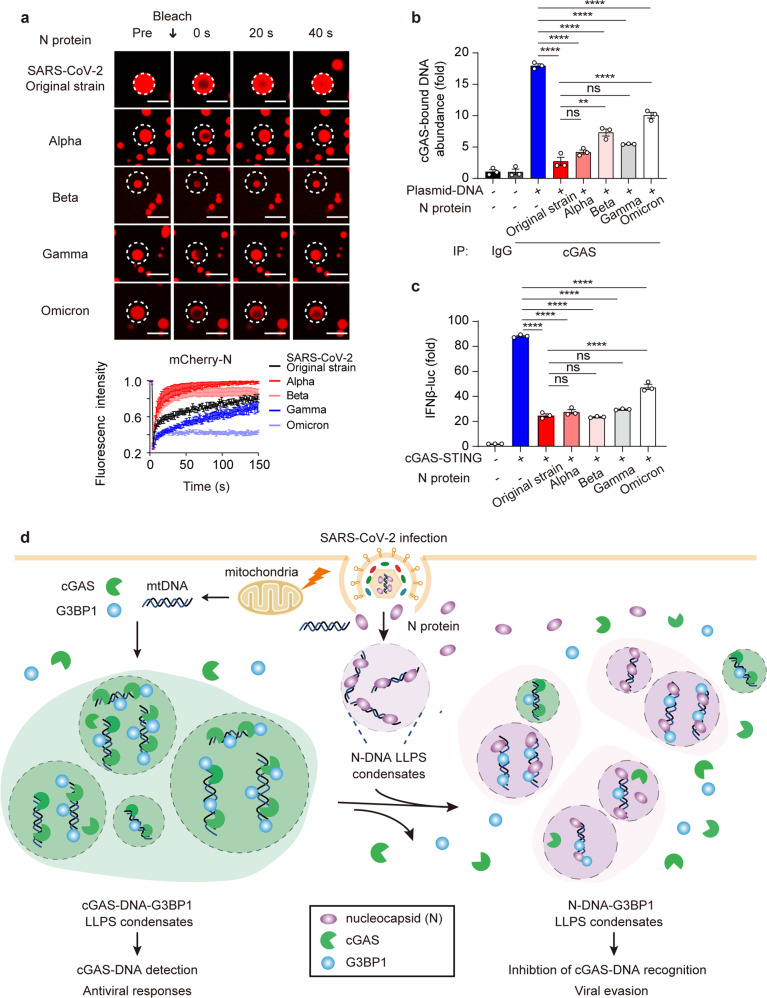
Nucleocapsid protein from SARS-CoV-2 and its different variants maintains the similar function on inhibiting cGAS-STING signaling. **a** Representative images of confocal microscopy showing recombinant mCherry-N protein from indicated variants (5 μM) mixed with ISD (2 μM) and incubated in LLPS buffer at 37 °C. Bleaching was performed at the indicated time points and the recovery occurred at 37 °C. Fluorescence intensity analysis of FRAP from *n* = 6 condensates over 150 s time course. Scale bars indicated 5 μm. **b** Calu3 cells expressed with N protein from SARS-CoV-2 and indicated variants were transfected with plasmid DNA (2 μg/mL) for 2 h before harvest. Cell lysates were collected, immunoprecipitated with A + G beads along with cGAS antibody, followed by qRT-PCR analysis of extracted DNA to detect cGAS-bound plasmid DNA (*mCherry*) abundance. **c** HEK-293T cells were transfected with IFNβ-luc, TK-luc, Flag-GFP-cGAS and HA-STING, together with plasmids that encoded N protein from indicated SARS-CoV-2 variants. Cell lysates were collected and luciferase assay was performed. **d** Schematic model depicting DNA-induced LLPS of N protein disrupting cGAS-G3BP1 LLPS condensation and cGAS DNA recognizing property. Data in **b**, **c** were expressed as mean ± SEM of 3 independent biological experiments. **P < 0.01, ****P < 0.0001, ns not significant (unpaired two-tailed student’s *t*-test). Similar results were obtained for 3 independent biological experiments in **a**

Different SARS-CoV-2 variants triggered different immune response.^4^ To further depict the relationship between LLPS ability and the function of N protein, we constructed and purified recombinant-N protein from Alpha (B.1.1.7), Beta (B.1.351), Gamma (P.1) and Omicron (B.1.1.529) variants and investigated their LLPS ability in vitro. We showed that N protein from original strain formed condensates with bigger size compared with that from SARS-CoV-2 Alpha, Beta, Gamma, and Omicron variant (Fig. 7a). Meanwhile, we checked the fluorescence recovery of N protein from different variants by FRAP. Intriguingly, the recovery of N protein condensates from SARS-CoV-2 original strain and the Alpha, Beta, and Gamma variants showed their high mobility, but the mobility of N protein from Omicron variant was dramatically decreased (Fig. 7a). By detecting the functions of N protein from original strain and different variants on cGAS-bound DNA abundance, we found that although all the N proteins from different variants exhibited the reduction on cGAS-bound DNA abundance, the function of N protein from Omicron variant was weaker compared with that from original strain or other variants (Fig. 7b). Moreover, IFNβ-luc experiment was performed to detect function of N protein from different variants on inhibiting cGAS-STING pathway. Consistent with the results on cGAS-bound DNA abundance, the luciferase results showed that N proteins from original strain or different variants possessed the ability to restrict cGAS-triggered activation of IFN-I signaling. However, compared with N proteins from original strain or Alpha, Beta, and Gamma variants, N protein from Omicron variant only showed slight recovery on the negative regulation on cGAS-STING-mediated signaling (Fig. 7c). These data together implied the crucial effect of LLPS of N protein in cGAS-induced IFN-I restriction, and demonstrated the evolved functions of different SARS-CoV-2 variants.

Taken together, we presented a schematic model for the function of DNA-induced N protein LLPS condensates in dismissing cGAS-G3BP1 condensation and impairing cGAS-DNA sensing (Fig. 7d). Invading SARS-CoV-2 triggers mitochondria dysfunction and the release of mtDNA. With the help of G3BP1, cytosolic DNA activates cGAS to form the LLPS condensates and facilitates the activation of IFN-I signaling. As counteractions, cytosolic DNA induces N protein LLPS and competitively binds with G3BP1 to disrupt the interaction as well as LLPS condensation between cGAS and G3BP1, consequently suppressing the cytosolic DNA recognition of cGAS as well as cGAS-mediated induction of IFN-I antiviral responses to promote immune evasion and enhance viral production (Fig. 7d).

## Discussion

COVID-19 is associated with respiratory illness, ARDS, along with the severe complications triggered by ARDS-related cytokine storm and tissue damage. The hyper-inflammatory state causes serious system disorder and dysfunction in host by mediating the reactive oxygen species (ROS) production^41^ The SARS-CoV-2-triggered ROS release and cell stress induces mitochondrial dysfunction in Huh7 cells, Vero E6 cells, and even endothelia cells, resulting in the depolarization of mitochondrial membrane, the opening of mitochondrial permeability transition pore and the increasing release of ROS, which may contribute on cell apoptosis or further cardiovascular complications or other in COVID-19,^27,28^ suggesting the significance of mitochondria in maintaining cellular oxidative homeostasis. However, the correlation between SARS-CoV-2 and mitochondria may be even more intricate. Given that SARS-CoV-2 RNA locates on both nucleus and mitochondria in host cells, SARS-CoV-2 might also hijack the mitochondria to conduct virus replication in this organelle.^28,42,43^ Here, we proved that SARS-CoV-2 invasion induced the damage of mitochondria as well as the release and accumulation of mtDNA in cytosol, subsequently triggering cGAS-STING pathway to activate IFN-I signaling. These results together demonstrated the crucial effect of mitochondria in virus replication and immune response.

cGAS is considered a striking antiviral molecule against RNA viruses. The ability of cGAS to recognize dsDNA depend on the length of DNA, which makes cGAS possible to detect misplaced cellular self-DNA triggered by RNA viruses-induced membrane rearrangement, as well as the exogenous DNA by infected pathogens, tissue destruction and cell damage,^10,13^ to induce IFN-I signaling and antiviral response.^17,20,21^ During COVID-19, SARS-CoV-2 infection might induce the induction of cGAS-STING pathway in lung epithelial cells in a RLR-independent manner.^18,44^ Moreover, cGAS-STING-mediated IFN-I signature is also prominent in COVID-19 patients with histological hallmarks of acute respiratory distress syndrome autopsy specimens.^19,44^ In the current study, we found IFN-I signaling was still activated to monitor virus infection in *RLRs*-deficient cells, and identified the involvement of cGAS in SARS-CoV-2-activated IFN-I responses by detecting mtDNA released from virus-induced damaged mitochondria. Given that cGAS work as an ISG, induction of cGAS expression by positive feedback of IFN-I signaling might also benefit the antiviral responses.^11^ Collectively, cGAS complementarily induced innate immune signaling by detecting accumulated mtDNA to amplify the antiviral responses along with the RLRs pathway, and uncovered that the participations of cGAS-STING pathway is required for full protection against RNA virus invasion. Due to the crucial antiviral functions of cGAS-STING axis, several cGAS-STING agonizts exhibited a more significant efficacy on SARS-CoV-2 infection than direct IFN treatment.^45,46^ Further investigation on cGAS agonizts might be conducted to benefit the COVID-19 therapy.

Considering the co-evolution between host and virus, it is not surprising that RNA viruses, such dengue virus, zika virus and other coronaviruses, have developed a series of sophisticated tactics to restrict cGAS-STING antiviral responses.^15,47,48^ For SARS-CoV-2, ORF3a, ORF9b and ORF10 proteins were shown to restrict the activation of IFN-I or cGAS-STING-mediated autophagy.^49–52^ Here, we found N protein disrupted the interaction between cGAS and its co-factor G3BP1, which was critical to cellular cGAS-DNA recognition, thereby restricting cGAS-DNA binding ability and cGAMP processing ability, to antagonize cGAS-STING pathway. We demonstrated a novel mechanism that SARS-CoV-2 utilized to restrict cGAS- triggered IFN-I signaling.

G3BP1 is the key component in stress granules. G3BP1/2 LLPS drives the formation of stress granules, which restricts protein synthesis and production of viral mRNAs to counter virus infection and replication.^53–55^ SARS-CoV-2 N protein could enter G3BP1 phase separation droplet and disassemble stress granule for viral productions during SARS-CoV-2 infection,^24,26,37,56^ and the binding of N protein with the SG-related amyloid proteins promotes their phase transition to aberrant amyloid aggregation.^57^ Besides its functions in stress granule, the involvement of G3BP1 in cGAS-DNA recognition has been identified, but the mechanism remains controversial. Cai et al. and Hu et al. proposed that cGAS was bound to G3BP1/stress granule along with DNA to form the LLPS condensates and then promoted the cGAS-DNA recognition,^35,36^ while Zhao et al. claimed that G3BP1 helped to form the cGAS pre-condensates but dissociated after DNA treatment.^58^ In the current work, we revealed the participation of G3BP1 in cGAS-DNA recognition when infected with SARS-CoV-2. In cell-free systems or *G3BP1*-deficient cells, N protein could not directly regulate the DNA detection function of cGAS. By competitively binding to NTF2L domain of G3BP1, N protein disrupted the cGAS-G3BP1 complex to limit the DNA recognition ability of cGAS and restricted the cGAS-mediated IFN-I signaling. Consistently, G3BP1 was also involved in the suppression of RLRs signaling by SARS-CoV-2 NSP5 and N protein.^56^ These results together depicted the crucial function of G3BP1 in anti-SARS-CoV-2 immune responses.

LLPS is a physiological phenomenon that regulates the generation of membraneless organelles, promotes protein-protein interaction, and accelerates enzyme activity. During SARS-CoV-2 infection, lots of biochemistry responses undergo LLPS to regulate immune signaling, antiviral response or viral replication. SARS-CoV-2 N protein has been reported to bind both RNA and DNA both in cell and in vitro.^59^ After interacting with nucleic acids, N protein is shown to undergo LLPS, thereby involved in various biological activities, including facilitating virion assembly, viral transcription, immune suppression as well as inflammatory hyperenhancement.^38,39,60,61^ Previous studies performed by us and other groups revealed the CTD domain is responsible for the RNA-triggered LLPS ability of N protein.^40,62^ Here, we discovered that N protein of SARS-CoV-2 could also undergo LLPS with cytosol DNA using its CTD domain. N protein formed the LLPS condensates together with G3BP1, consequently restricting the formation of cGAS-G3BP1 condensates, reducing the recognition and LLPS of cGAS-DNA. N protein △CTD mutant lost its capability to interact with G3BP1, and no longer inhibited cGAS-DNA detection or reduced the cGAS-STING signaling. Therefore, our findings uncovered the physiological function of DNA-induced LLPS of N protein in regulating antiviral response. Given the importance of N protein LLPS properties in restricting antiviral response and viral immune evasion, future works should be performed to explore novel strategies against SARS-CoV-2 infection by targeting the LLPS of N protein.

## Materials and methods

### Cell Culture

Calu3 cells were incubated in 37 °C chamber (Thermo Fisher Scientific) with 5% CO_2_ using MEM with 10% fetal bovine serum, 1% NEAA, 1 mM Sodium Pyruvate and 1% L-glutamine. MEM, fetal bovine serum, NEAA, Sodium Pyruvate and L-glutamine was bought from Gibco. Vero E6 cells, HEK-293T cells, HeLa cells A549 cells and Huh7 cells were cultured in DMEM with 10% fetal bovine serum and 1% L-glutamine and incubated in 37 °C chamber with 5% CO_2_. DMEM was brought from Corning. Detailed product catalogs are listed in Supplementary Table 1.

### Cell lines generation

N protein stable or inducible expressing A549 and Calu3 cell lines, ACE2-expressing A549 cell line were constructed in our previous studies,^40,63^ while cGAS stable expressing HEK-293T cell line and *cGAS*^sgRNA^ Calu3 cell line were constructed with lentiviral system. In short, indicated lentiviral were generated in HEK-293T cells by transfecting plenti-EH-N protein-2F, plenti-TRE-N protein-2F plasmid, plenti-EH-ACE2-2F, plenti-EH-cGAS-2F or sg-cGAS-pLentiCRISPR V2 along with vectors encoding pLP1, pLP2, and Plp/VSV-G. HEK-293T cells, Teton-3G-expressing Calu3 and A549 cells were infected with lentivirus to construct indicated cell lines. Puromycin (concentration up to 3 μg/mL) was used to screen puromycin-resistant cells. After screening, cells were used for following experiments. The indicated small-guide RNA (sgRNA) sequences were synthesized by Sangon Biotech. sgRNA sequence is listed in Supplementary Table 2.

### Viruses

SARS-CoV-2 (Accession number: MT123290) virus generation and titer measurement were performed in Vero E6 cells, as previously described.^40,64^ After stimulating with SARS-CoV-2 virus, the cells were gently shaken every 15 min within 2 h, then changed to fresh culture medium and cultured at 37 °C. Calu3 cells were infected with indicated MOI and time points.

### Antibodies and reagents

Anti-Flag-Horseradish peroxidase (HRP), anti-β-actin and anti-cGAS were purchased from Sigma-Aldrich. Anti-hemagglutinin (HA)-HRP and anti-c-Myc-HRP were purchased from Roche Applied Science. Anti-DNA was purchased from Millipore. Anti-phospho-STAT1 (Tyr701), anti-STAT1, anti-phospho-TBK1 (Ser172), anti-TBK1, anti-phospho-IRF3 (Ser396) and anti-IRF3 were purchased from Cell Signaling Technology. Anti-G3BP1, anti-TOM20, anti-PARP and anti-caspase3 were purchased from Proteintech. Anti-SARS-CoV-2-N protein was purchased from Sino Biological. Goat anti-Rabbit IgG (H + L) cross-adsorbed secondary antibody, HRP, goat anti-Mouse IgG (H + L) cross-adsorbed secondary antibody, HRP, goat anti-Mouse IgG (H + L) highly cross-adsorbed secondary antibody, Alexa Fluor 568, goat anti-Mouse IgG (H + L) highly cross-adsorbed secondary antibody, Alexa Fluor 488, goat anti-Rabbit IgG (H + L) cross-adsorbed secondary antibody, Alexa Fluor 568 and goat anti-Rabbit IgG (H + L) highly cross-adsorbed secondary antibody, Alexa Fluor 488 were purchased by Invitrogen. Detailed antibody catalogs are listed in Supplementary Table 1.

Protein A agarose, protein G agarose and NeutrAvidin agarose resin were purchased from Pierce. Anti-Flag Gel, HT-DNA, DTT and imidazole were purchased from Sigma-Aldrich. Protease inhibitors and phosSTOP phosphatase inhibitor cocktail were purchased from Roche Applied Science. Isopropyl-beta-D-thiogalactopyranoside (IPTG), superluminal™ high-efficiency transfection reagent, preStain™ protein marker and 30% Acr/Bis (29:1) were purchased from MIKX. Detailed product catalogs are listed in Supplementary Table 1.

Cy5-ISD, biotin-ISD, ISD, Cy5-100 bp dsDNA and 100 bp dsDNA were synthesized by Sangon Biotech. Sequences are listed in Supplementary Table 2.

### Plasmids

SARS-CoV-2 proteins plasmids were chemically synthesized based on SARS-CoV-2 sequence, and generously given by Pei-Hui Wang lab as previously described.^40,64^ Nucleocapsid (N) protein was subcloned into plenti-TRE-DEST-2F or plenti-EH-DEST-2F vector for inducible or stably expressing system by standard PCR techniques for construction of cell lines. Plasmid with IFNβ-luciferase reporter (firefly luciferase; 80 ng) and pRL-TK luciferase reporter (Renilla luciferase plasmid; 10 ng) was previously described.^65^ cGAS, STING and G3BP1 were cloned by standard PCR techniques from cDNA and constructed into pcDNA3.1 with HA, Flag, or Myc tags or fluorescent protein expressing vectors. SARS-CoV-2 N (full length, FL), N-△NTD (amino acid residues 44–180 deletion mutant), N-△SR (amino acid residues 181–246 deletion mutant) and N-△CTD (amino acid residues 247–364 deletion mutant) were subcloned into pcDNA3.1-HA, Flag, Myc or indicated fluorescent protein expressing vectors for mammalian cells expression. We used pET-28a vector to generate the expressing plasmids encoding N-FL, N-△NTD, N-△SR, N-△CTD, G3BP1 and cGAS conjugated mCherry, CFP or GFP tag to conducted the recombinant proteins purification in vitro. Fast site-directed mutagenesis Kit was employed to generate plasmids encoding N protein from different SARS-CoV-2 variants Fast site-directed mutagenesis Kit (TIANGEN). Detailed product catalogs are listed in Supplementary Table 1.

### Luciferase and reporter assays

HEK-293T cells were transfected with plasmids encoding the IFNβ-luc and TK-luc and plasmids encoding indicated protein. After treating with indicated stimulation, cells were then collected with passive lysis buffer (Promega). Enzyme activity of IFNβ-luc was measured and normalized to Renilla luciferase activity measured by TK-luc using Dual-Luciferase® reporter assay system (Promega). Fold induction level relative to the basal level was analyzed and showed. Detailed product catalogs are listed in Supplementary Table 1.

### In vitro recombinant protein expression and purification

As previous described,^40^ the expressing plasmids encoding N-FL, N-△NTD, N-△SR, N-△CTD, cGAS, and G3BP1 conjugated mCherry, GFP or CFP tag were transformed into BL21 *E.coli*. *E.coli* with expressing plasmids were cultured in LB with Kanamycin (50 µg/ml) at 37 °C for about 12 h till OD600 = 0.6. 1 mM IPTG was added to induced protein expression at 37 °C for 8 h, then the *E. coli* were collected by centrifugation at 4000 rpm, 4 °C, 10 min and resuspended in lysis buffer. Cells were lysed by sonication on ice and then centrifuged at 12,000 rpm, 4 °C, 30 min to remove debris and collected the supernatant. The supernatant was treated with Benzonase (Millipore) to exclude nucleus acid, and incubated with Ni-NTA agarose beads (QIAGEN) 4 °C overnight. Ni-NTA beads were then rinsed with wash buffer, and proteins were eluted with elution buffer. Dialysis experiments were conducted for purified proteins by PD10 desalting column (GE Healthcare) to exclude high concentration of salt, followed by concentrating with storage buffer using Amicon Ultra 30 K at 5000 rpm, 4 °C, 15 min. Protein concentration were quantified by the BCA method (Pierce), verified the 260/280 (under 0.55 to make sure the nuclear-acid was excluded) and stored at −80 °C. Detailed product catalogs are listed in Supplementary Table 1. Detailed reagent formulations are listed in Supplementary Table 3.

### RNA extraction and quantitative real-time PCR

Total RNA from whole cells and SARS-CoV-2 RNA from virus-infected cells supernatant were extracted by TRIzol reagent (Invitrogen), followed with cDNA generation with HiScript® III RT SuperMix for qPCR (+gDNA wiper) (Vazyme). Quantitative real-time PCR (qRT-PCR) was performed in Roche LightCycler 480 System (Basel, Switzerland) by using the 2 × PolarSignal™ SYBR Green mix Taq (MIKX). Total RNA sample from whole cells were normalized to *RPL13A* expression. SARS-CoV-2 RNA samples from virus-infected cells supernatant were quantified with addition of 10 ng *mCherry* DNA and normalized to *mCherry* DNA abundance. Detailed product catalogs are listed in Supplementary Table 1 and primer sequences are listed in Supplementary Table 2.

### Quantification of cytosolic DNA abundance and cGAS-bound DNA abundance

For the cytosolic DNA analysis, cytosolic lysates were collected with procedures used in our previous study.^66^ To avoid artificial mtDNA release, the cells were collected by centrifugation at 800 × *g* 5 min at 4 °C, and cell pellets was resuspended and lysed with cytosolic extraction buffer by incubated at 4 °C, 10 min. The supernatant of cytosolic fraction was collected with centrifugated at 10,000 × *g*, 4 °C, 15 min.

For cGAS-bound DNA analysis, cells were stained with 1% formaldehyde and incubated at room temperature for 10 min, then mixed with 10% glycine from 1.375 M stock. Cells were rinsed 3 times with ice-cold PBS, collected and lysed with low salt lysis buffer (LSB). After sonicated 9 times for 10–20 s at 80% setting in sonicator, the extracts were centrifuged for 1000 rpm, 4 °C for 5 min. Supernatants were collected, co-incubated with A + G beads together with IgG or cGAS antibody to pull-down cGAS by constant rotation at 4 °C overnight to collect cGAS-bound DNA. The mixtures were centrifuged and the beads were rinsed 5 times with ice-cold LSB. 200 μL elution buffer were added to beads and incubated at 65 °C for 10 min. Centrifuged to collect the supernatant and elute the beads again and combined the eluates. 21 μL NaCl from 4 M stock were added to IP samples and incubated at 65 °C for 4 h. Next, 1 μL RNase A from 10 mg/mL stock were added to samples and incubated at 37 °C for 1 h, followed with adding 4 μL 0.5 M EDTA and 2 μL 10 mg/mL proteinase K and incubated at 42 °C for 2 h. Detailed reagent formulations are listed in Supplementary Table 3.

Extraction and analysis of cytosolic DNA or cGAS-bound DNA were performed by phenol/chloroform/isoamyl alcohol assay, followed with qRT-PCR assay. Primer sequences as previous used are listed in Supplementary Table 2.^15,66^

### Quantification of cellular IFNβ and cGAMP production

Calu3 cells infected with SARS-CoV-2 were collected with mPER lysis buffer (Thermo Fisher) and inactivated SARS-CoV-2 followed with UV radiation. The production of IFNβ in cells was measured by LumiKine™ Xpress mIFN-β 2.0 (Invivogen) was used according to the manufacturer instructions. The production of cGAMP in cells was measured by 2'3’-cGAMP ELISA Kit (Cayman Chemical) according to the manufacturer instructions. Detailed product catalogs are listed in Supplementary Table 1.

### Quantification of cGAMP processing ability in vitro

cGAMP processing ability of cGAS in vitro was measured with Kinase-Glo® Luminescent Kinase Assay (Promega). Detailed product catalog is listed in Supplementary Table 1.

### RNA interference

Nucleotide siRNA duplexes were synthesized from Sangon Biotech. RNAiMAX (Invitrogen) was used to transfect siRNA to cells. Detailed product catalog is listed in Supplementary Table 1 and RNA oligonucleotides used in this study are listed in Supplementary Table 2.

### Immunoprecipitation (IP) and immunoblotting analysis

Whole cell lysates (WCL) were collected with LSB buffer after treating with indicated and appropriate stimulation. For IP experiments, WCL was incubated with anti-Flag agarose gels or A + G beads together with indicated antibodies at 4 °C, overnight. Beads were then rinsed 3–5 times with ice-cold LSB, eluted with 2 × SDS Loading Buffer at 100 °C followed with SDS-PAGE experiments. Proteins were transferred to PVDF membranes (Bio-Rad), blocked with skim milk, and then incubated with the indicated antibodies. Proteins were detected by using Immobilon Western Chemiluminescent HRP Substrate (Millipore). Detailed product catalogs are listed in Supplementary Table 1.

### Immunoprecipitation analysis of cGAS-bound biotin-DNA

N protein-inducible Calu3 cells were stimulated with biotin-ISD (2 μg/mL) for 2 h before harvest of WCL. The WCL incubated with NeutrAvidin Agarose Resin at 4 °C, overnight. Beads were then rinsed 3–5 times with ice-cold LSB and followed with immunoblotting assay.

### Fluorescence microscopy

Glass Bottom culture dishes (Nest Scientific) were used to culture cells for fluorescence experiments. After treated with indicated stimulation, cells were fixed by 4% paraformaldehyde for 10 min, followed with 3 times wash with PBS. Cells were then permeabilized with methyl alcohol for 30 min at −20 °C and rinsed with PBS for 3 times. After blocking in 6% goat serum for 1 h at room temperature, cells were incubated with primary antibodies at 4 °C, overnight. PBSt (PBS with 0.1% Tween20) was used to wash the cells for 3 times and subsequently incubated with fluorescently labeled secondary antibodies at room temperature for 1 h. Confocal images were obtained by microscope (TCS SP8 STED 3X, Leica) equipped with 100 × 1.40 NA oil objectives. The images were processed for gamma adjustments in Leica AS Lite. Indicated data including the number of condensates were counted and analyzed by ImageJ software (National Institutes of Health, https://imagej.net/software/imagej/).

### In vitro LLPS assay

The purified recombinant proteins were mixed at indicated concentration with LLPS buffer and 5% PEG8000, followed by incubating with indicated dsDNA for 5 min at 37 °C. The mixture was pipetted onto glass bottom dish and imaged by microscope (TCS SP8 STED 3X, Leica) equipped with 100 × 1.40 NA oil objectives. Detailed reagent formulations are listed in Supplementary Table 3.

### Fluorescence recovery after photobleaching (FRAP)

FRAP assay was conducted by Leica TCS SP8 STED 3X confocal microscopy. 488-nm or 568-nm laser beam at 100% laser power was used to bleach the fluorescent protein at a region of interest (ROI), followed with collecting time-lapse images. Fluorescence intensity of indicated ROI was measured and normalized to the fluorescence intensity of pre-bleaching image by Leica AS Lite. The FRAP results were analyzed by GraphPad Prism.

### Statistical analyses

Statistical analyses were conducted by GraphPad Prism (GraphPad Software, Inc, La Jolla, CA, USA). Differences of means ± SD/SEM were tested for statistical significance with unpaired two-tailed Student’s *t* test. *p < 0.05, **p < 0.01, ***p < 0.001, ****p < 0.0001; ns not significant.

## Supplementary information

Supplementary information

Supplementary Materials for uncropped figure

## Data Availability

All data supporting the findings of this study are available within the article and its supplementary information or from the corresponding author upon reasonable request.
