# Supplementary material for: Phase-separated nucleocapsid protein of SARS-CoV-2 suppresses cGAS-DNA recognition by disrupting cGAS-G3BP1 complex
Source: Signal Transduct Target Ther. 2023 Apr 26;8:170. doi: 10.1038/s41392-023-01420-9 (PMC10131525; doi:10.1038/s41392-023-01420-9)
Supplement: Supplementary file 1 — Supplementary information [file 41392_2023_1420_MOESM1_ESM.docx]

Supplementary Materials for

**Phase-separated nucleocapsid protein of SARS-CoV-2 suppresses cGAS-DNA recognition by disrupting cGAS-G3BP1 complex**

Sihui Cai, Chenqiu Zhang, Zhen Zhuang, Shengnan Zhang, Ling Ma, Shuai Yang, Tao Zhou, Zheyu Wang, Weihong Xie, Shouheng Jin, Jincun Zhao, Xiangdong Guan, Jianfeng Wu, Jun Cui, Yaoxing Wu

Correspondence to: Yaoxing Wu (wuyaox5@mail.sysu.edu.cn), Xiangdong Guan (guanxd@mail.sysu.edu.cn), Jianfeng Wu (wujianf@mail.sysu.edu.cn) or Jun Cui (cuij5@mail.sysu.edu.cn)

**This PDF file includes:**

Supplementary Fig. 1 to 6

Supplementary Table 1 to 3

Supplementary Figures


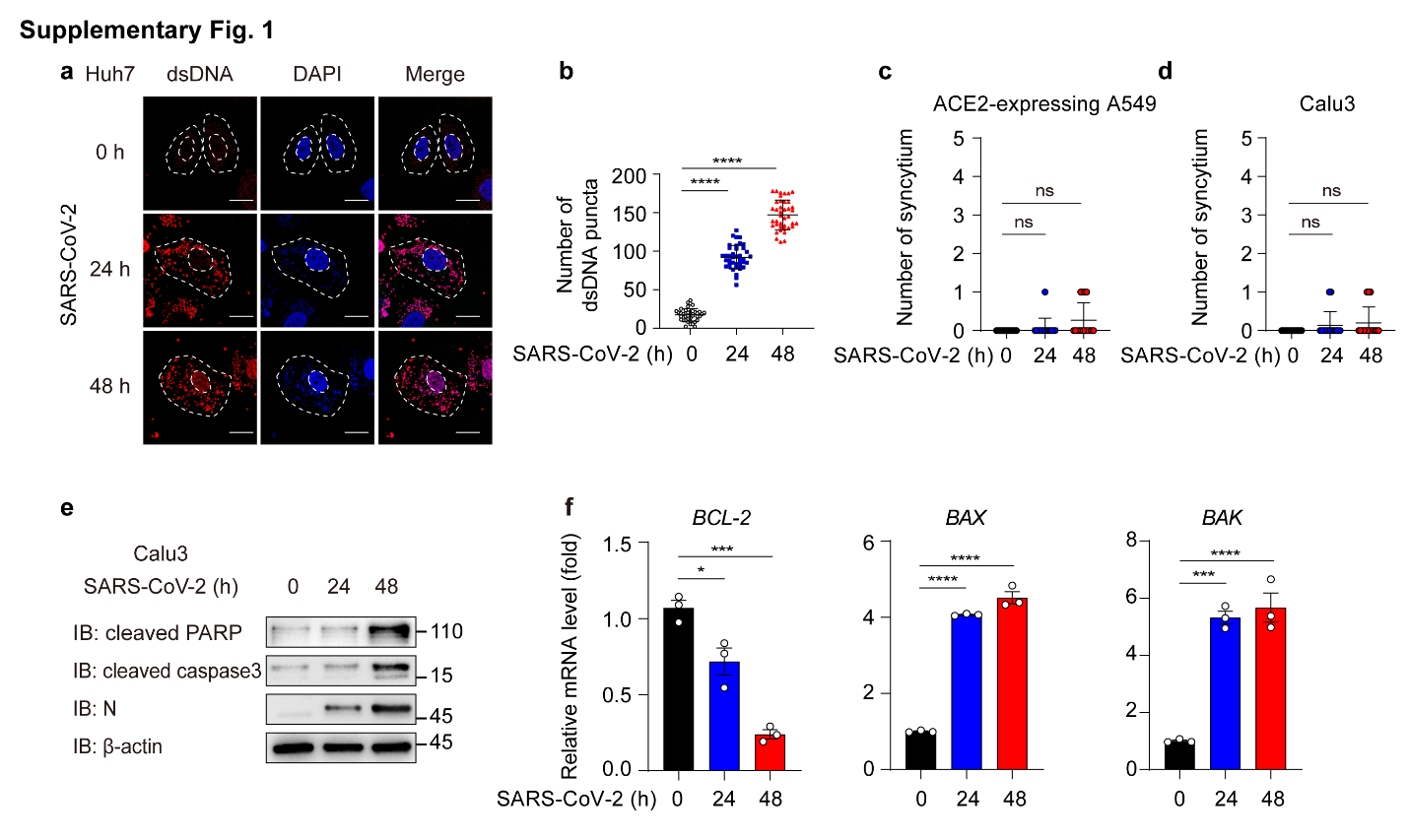


**Supplementary Fig. 1 SARS-CoV-2 induces mitochondrial apoptosis and mtDNA accumulation.**

(**a-b**) Representative confocal micrographs of Huh7 cells infected with SARS-CoV-2 (MOI=0.1) for indicated time points, followed by labeled double-strand DNA (dsDNA, red) (**a**). Dotting lines indicated the shape of cells. Scale bars indicated 10 μm. The number of dsDNA puncta per cell (**b**) was measured and analyzed from n=20 cells.

(**c-d**) Number of syncytia of 10 views per cell of 20 ACE2-expressing A549 cells (**c**) or Calu3 cells (**d**) infected with SARS-CoV-2 (MOI=0.1) for indicated time points.

(**e**) Calu3 cells were infected with SARS-CoV-2 (MOI=0.1) for indicated time points. Cell lysates were collected and immunoblot assay was performed.

(**f**) Quantitative real-time PCR (qRT-PCR) with reverse transcription analysis of *BCL-2*, *BAX* and *BAK* mRNA of Calu3 cells infected with SARS-CoV-2 (MOI=0.1) for indicated time points.

Data in (**b**) were expressed as mean ± SD of 20 cells for each condition. Data in (**c-d**) were expressed as mean ± SD of 20 cells for each condition. Data in (**f**) were expressed as mean± SEM of 3 independent biological experiments. *P<0.5, **P<0.01, ***P<0.001, ****P<0.0001, ns, not significant (unpaired two-tailed student’s *t*-test). Similar results were obtained for 3 independent biological experiments in (**a** and **e**).


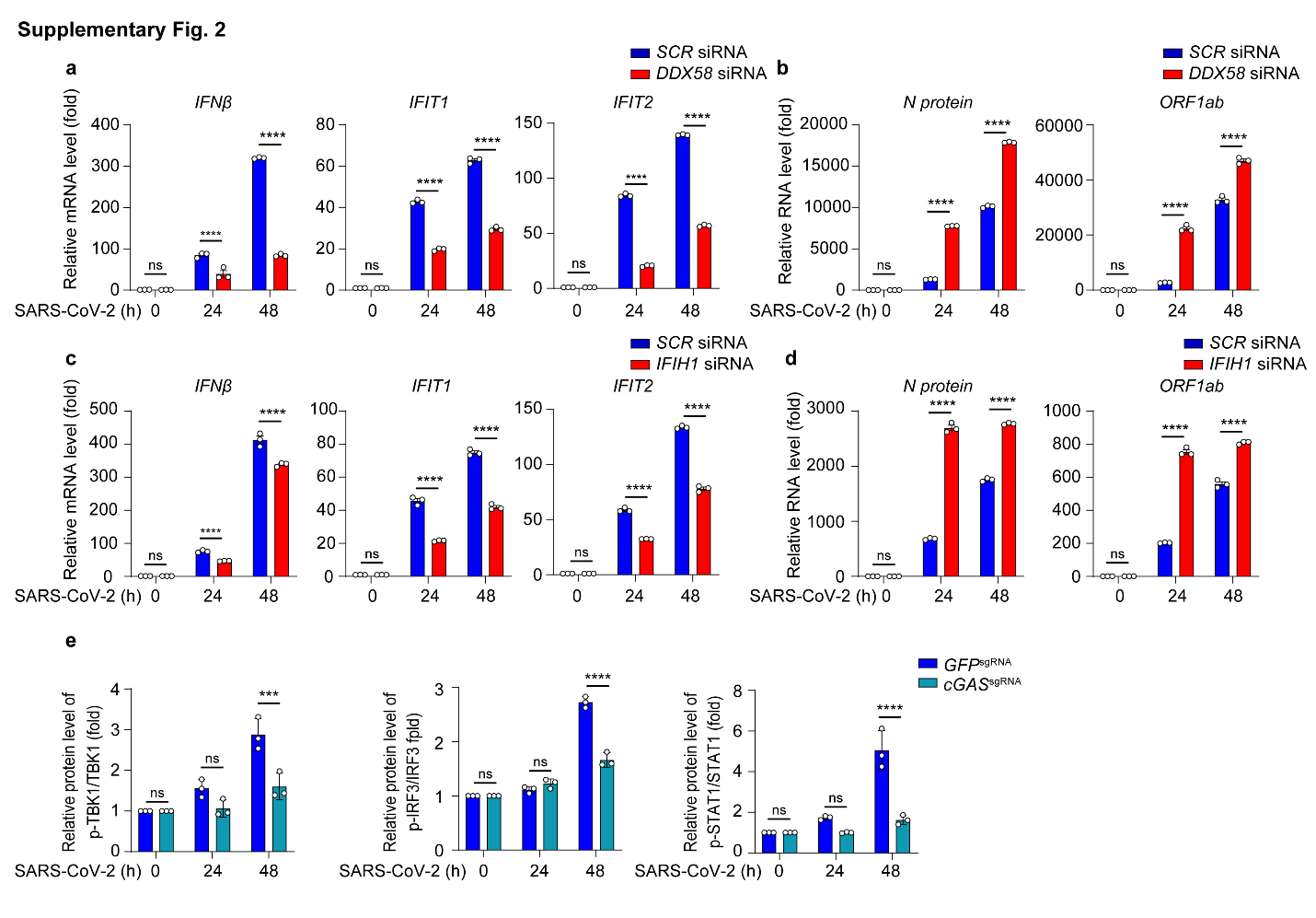


**Supplementary Fig. 2 SARS-CoV-2 induces RLRs-independent IFN-I signaling.**

(**a**) qRT-PCR with reverse transcription analysis of *IFNβ*, *IFIT1* and *IFIT2* mRNA of Calu3 cells transfected with *scramble* (*SCR*) siRNA or *DDX58* siRNA along with SARS-CoV-2 (MOI=0.1) infection for indicated time points.

(**b**) qRT-PCR with reverse transcription analysis of SARS-CoV-2 *N protein* and *ORF1ab* RNA of Calu3 cells transfected with *scramble* (*SCR*) siRNA or *DDX58* siRNA along with SARS-CoV-2 (MOI=0.1) infection for indicated time points.

(**c**) qRT-PCR with reverse transcription analysis of *IFNβ*, *IFIT1* and *IFIT2* mRNA of Calu3 cells transfected with *scramble* (*SCR*) siRNA or *IFIH1* siRNA along with SARS-CoV-2 (MOI=0.1) infection for indicated time points.

(**d**) qRT-PCR with reverse transcription analysis of SARS-CoV-2 *N protein* and *ORF1ab* RNA of Calu3 cells transfected with *scramble* (*SCR*) siRNA or *IFIH1* siRNA along with SARS-CoV-2 (MOI=0.1) infection for indicated time points.

(**e**) Quantification of the protein level of pTBK1/TBK1, pIRF3/IRF3 and p-STAT1/STAT1 in Fig. 2a.

Data in (**a-d**) were expressed as mean values ± SEM of 3 independent biological experiments. Data in (**e**) were expressed as mean ± SD of 3 independent biological experiments. ***P<0.001, ****P<0.0001, ns, not significant (unpaired two-tailed student’s *t*-test).


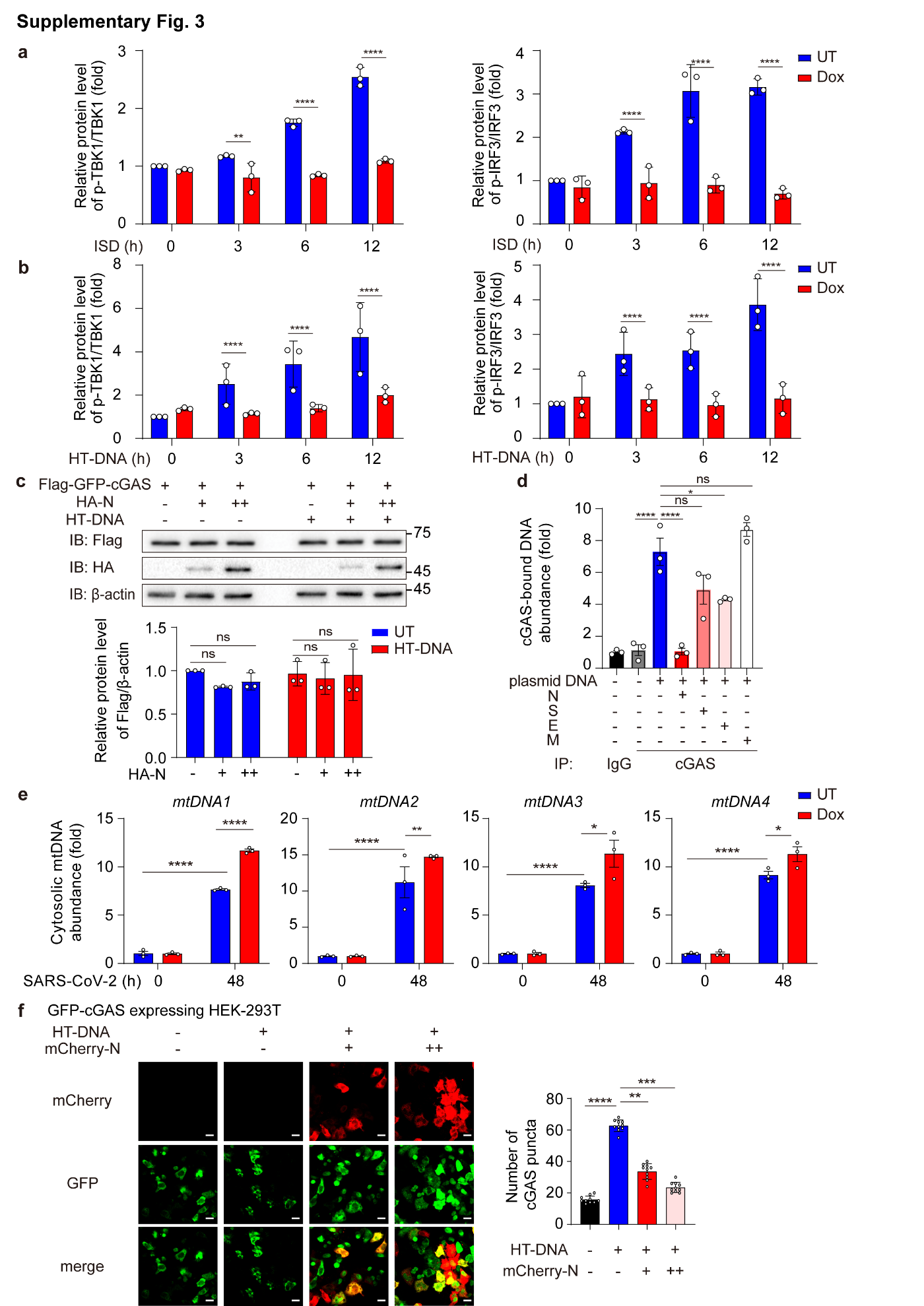


**Supplementary Fig. 3 SARS-CoV-2 N protein reduces the DNA recognition of cGAS.**

(**a**) Relative protein level of pTBK1/TBK1, pIRF3/IRF3 in Fig. 3d.

(**b**) Relative protein level of pTBK1/TBK1, pIRF3/IRF3 in Fig. 3e.

(**c**) HEK-293T cells were transfected with Flag-GFP-cGAS and increasing concentration of HA-N protein, followed with HT-DNA (2 μg/mL) stimulation for 12 hours or left untreated (UT). Cell lysates were collected and immunoblot analysis was performed. Relative protein level of Flag/β-actin was measured and showed.

(**d**) HEK-293T cells expressing SARS-CoV-2 N protein, S protein, E protein or M protein were transfected with mCherry plasmid (2 μg/mL) as plasmid DNA for 2 hours before harvest. Cell lysates were collected, immunoprecipitated with A+G beads together with cGAS antibody, followed by qRT-PCR analysis of extracted DNA to detect cGAS-bound plasmid DNA (*mCherry*) abundance.

(**e**) qRT-PCR of cytosolic mtDNA abundance of N protein-inducible Calu3 cells treated with Dox (200 ng/mL) or left untreated (UT) were infected by SARS-CoV-2 (MOI=0.1) for indicated time points.

(**f**) Representative images of GFP-cGAS-expressing HEK-293T cells transfected with mCherry-N followed with HT-DNA (2 μg/mL) stimulation for 12 hours. The number of GFP-cGAS puncta was counted and analyzed.

Data in (**a-c**) were expressed as mean ± SD of 3 independent biological experiments. Data in (**d, e**) were expressed as mean values ± SEM of 3 independent biological experiments. Data in (**f**) are expressed as mean ± SD of 10 cells for each condition. *P<0.5, **P<0.01, ***P<0.001, ****P<0.0001, ns, not significant (unpaired two-tailed student’s *t*-test).


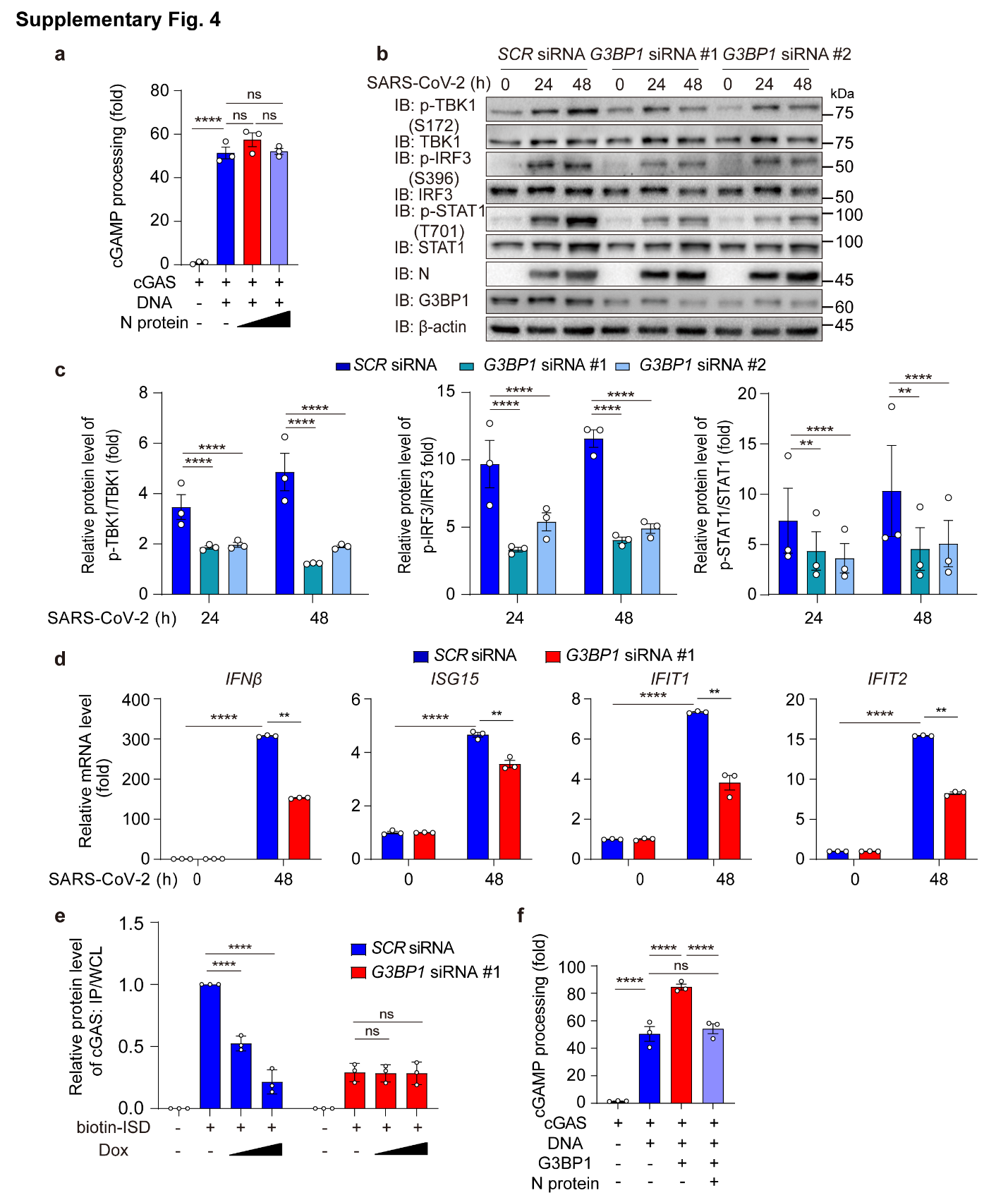


**Supplementary Fig. 4 G3BP1 is crucial for SARS-CoV-2-induced IFN-I signaling and the function of N protein.**

(**a**) *In vitro* cGAMP processing ability of recombinant cGAS (10 μM) incubated with recombinant N protein (10 μM) and ISD (2 μM).

(**b**) Calu3 cells transfected with *scramble* (*SCR*) siRNA, *G3BP1* siRNA #1 or *G3BP1* siRNA #2 were infected by SARS-CoV-2 (MOI=0.1) for indicated time points. Cell lysates were collected and immunoblot analysis was performed.

(**c**) Relative protein level of pTBK1/TBK1, pIRF3/IRF3 and p-STAT1/STAT1 in Supplementary Fig. 4b.

(**d**) qRT-PCR with reverse transcription analysis of *IFNβ*, *ISG15*, *IFIT1* and *IFIT2* mRNA of Calu3 cells transfected with *scramble* (*SCR*) siRNA, *G3BP1* siRNA #1 or *G3BP1* siRNA #2 followed with SARS-CoV-2 (MOI=0.1) infection for indicated time points.

(**e**) Relative protein level of cGAS: IP/WCL in Fig. 4b.

(**f**) *In vitro* cGAMP processing ability of recombinant cGAS (10 μM) incubated with recombinant G3BP1 (10 μM), recombinant N protein (10 μM) and ISD (2 μM).

Data in (**a, d**, **f**) were expressed as mean values ± SEM of 3 independent biological experiments. Data in (**c, e**) were expressed as mean ± SD of 3 independent biological experiments. **P<0.01, ****P<0.0001, ns, not significant (unpaired two-tailed student’s *t*-test). Similar results were obtained for 3 independent biological experiments in (**b**).


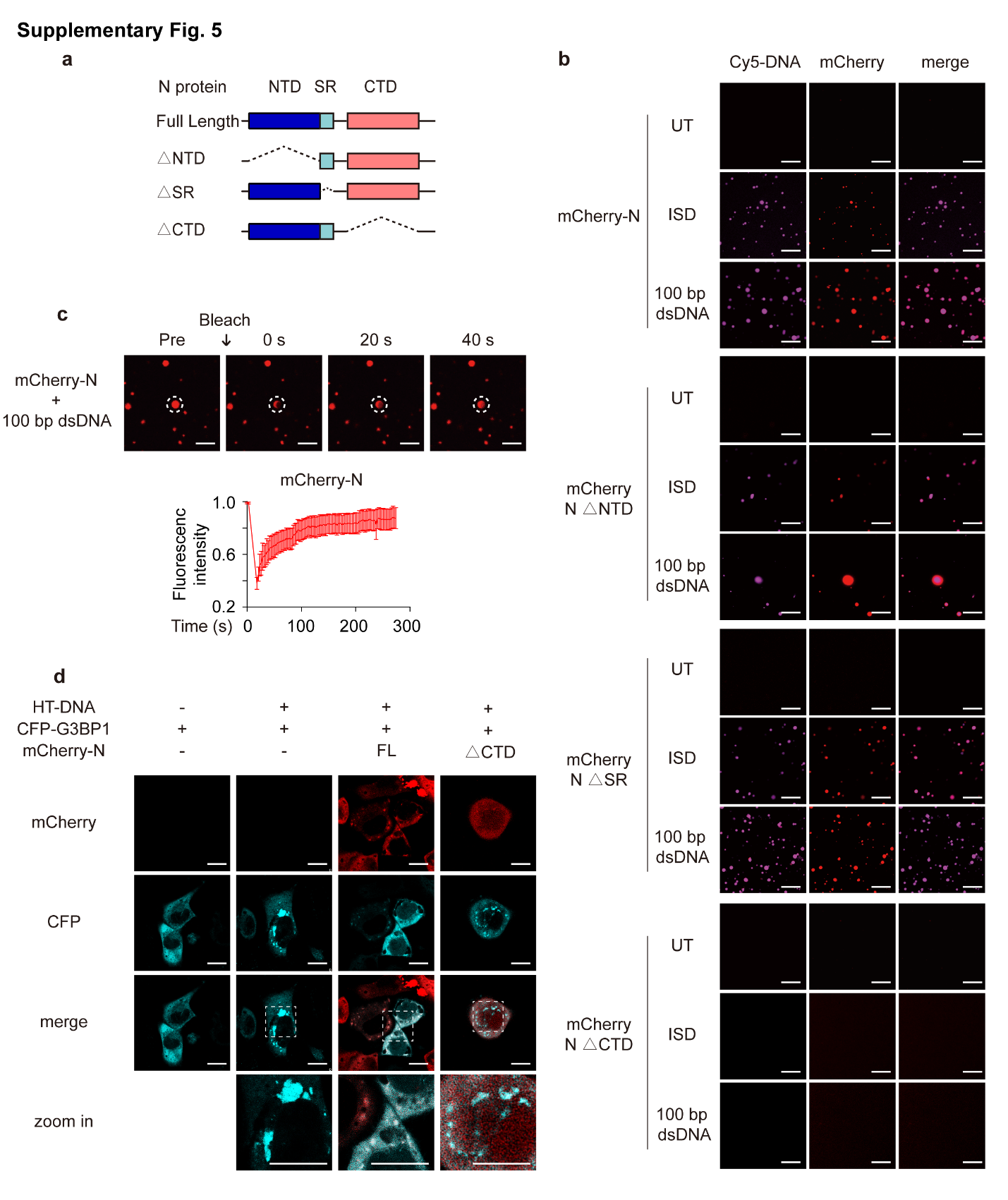


**Supplementary Fig. 5 SARS-CoV-2 N protein undergoes DNA-induced LLPS *in vitro*.**

(**a**) Schematic figure of domain organization of SARS-CoV-2 N protein and its domain deletion mutant.

(**b**) Representative images of confocal microscopy of recombinant N protein mCherry-N protein (10 μM), △NTD (10 μM), △SR (10 μM) or △CTD (10 μM) mixed with Cy5- ISD (2 μM, magenta) and incubated in LLPS buffer at 37°C. Scale bars indicated 5 μm.

(**c**) Live cell images of recombinant N protein mCherry-N protein (10 μM) mixed with Cy5- ISD (2 μM, magenta) and incubated in LLPS buffer at 37°C. Scale bars indicated 10 μm. Fluorescence intensity analysis of fluorescence recovery after photobleaching (FRAP) over a 90 seconds time course from 7 droplets.

(**d**) Representative images of confocal microscopy of HeLa cells expressing CFP-G3BP1 transfected with mCherry-N or mCherry-N-△CTD, along with HT-DNA (2 μg/mL) stimulation for 12 hours. Scale bars indicated 5 μm.

Similar results were obtained for 3 independent biological experiments in (**b-d**).


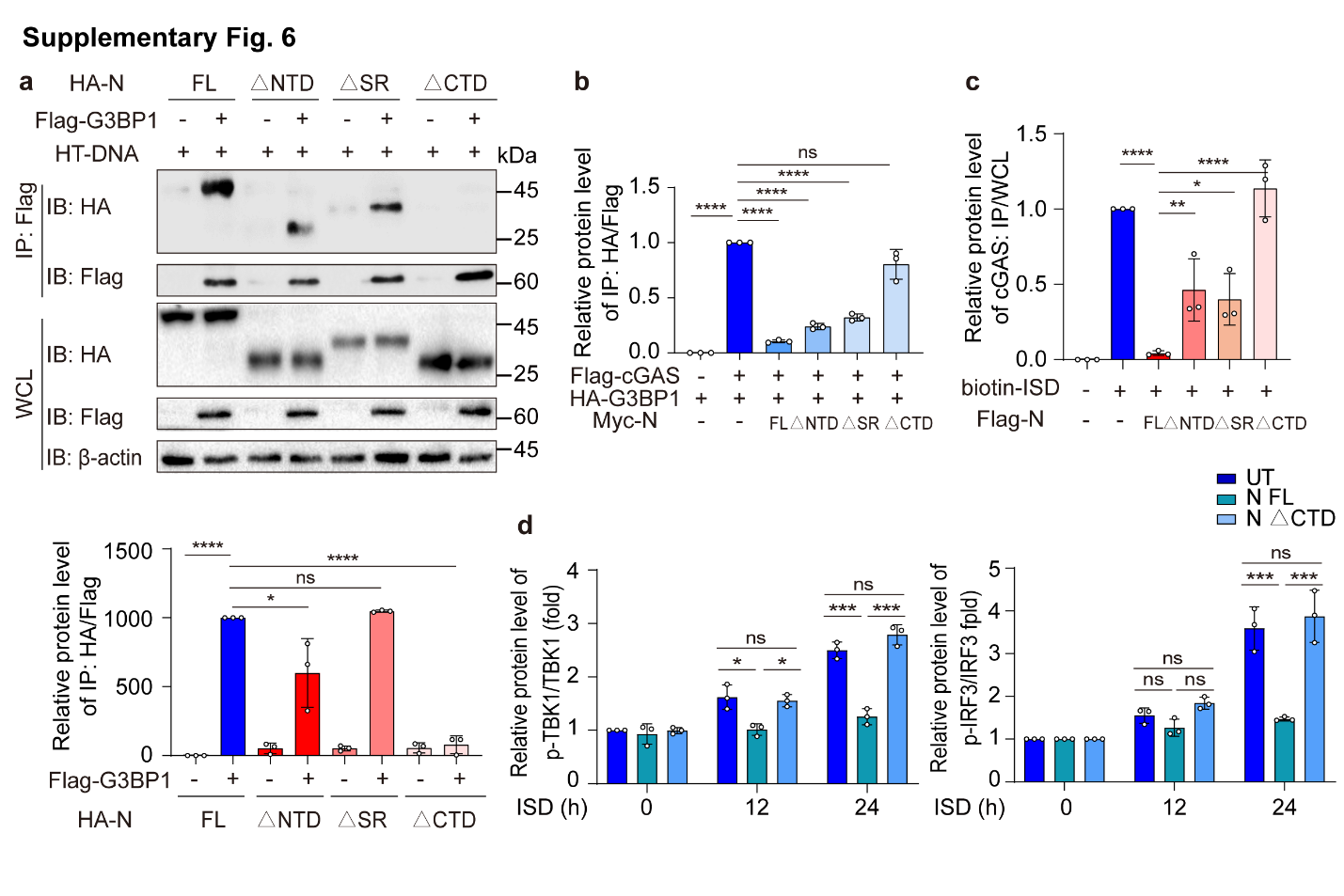


**Supplementary Fig. 6 SARS-CoV-2 N protein LLPS ability related-CTD is responsible for the interaction with G3BP1 and reduction on cGAS-STING pathway.**

(**a**) HEK-293T cells expressing Flag-G3BP1, along with HA-N full-length (FL), △NTD, △SR or △CTD with HT-DNA (2 μg/mL) treatment for 2 hours. Cell lysates were collected, immunoprecipitated with HA-beads, followed by immunoblotting analysis. Relative protein level of IP: HA/Flag was measured and showed.

(**b**) Relative protein level of IP: HA/Flag in Fig. 6a.

(**c**) Relative protein level of cGAS: IP/WCL in Fig. 6b.

(**d**) Relative protein level of p-TBK1/TBK1 and p-IFR3/IRF3 in Fig. 6e.

Data in (**a-d**) were expressed as mean ± SD of 3 independent biological experiments. *P<0.05, **P<0.01, ***P<0.001, ****P<0.0001, ns, not significant (unpaired two-tailed student’s *t*-test).

**Supplementary Table 1 Catalogs of reagents and antibodies.**

**Reagents**

| **Reagent** | **Source** | **Identifier** |
| --- | --- | --- |
| MEM | Gibco | 11095-080 |
| NEAA | Gibco | 11140050 |
| Sodium Pyruvate | Gibco | 11360070 |
| Fetal Bovine Serum | Gibco | 10099 |
| L-glutamine | Gibco | 35050061 |
| DMEM | Corning | 10-013-CV |
| Protein A agarose | Pierce | 20333 |
| Protein G agarose | Pierce | 20399 |
| NeutrAvidin agarose resin | Pierce | 29200 |
| BCA method | Pierce | 23250 |
| Anti-Flag (M2) Gel | Sigma-Aldrich | A2220 |
| HT-DNA | Sigma-Aldrich | 438545-06-3 |
| DTT | Sigma-Aldrich | 10197777001 |
| Imidazole | Sigma-Aldrich | 56750 |
| Protease inhibitors | Roche Applied Science | 11697498001 |
| phosSTOP phosphatase inhibitor cocktail | Roche Applied Science | 4906837001 |
| Isopropyl-beta-D-thiogalactopyranoside (IPTG) | MIKX | CA413 |
| Superluminal™ high-efficiency transfection reagent | MIKX | 11231804 |
| PreStain™ protein marker | MIKX | DB180 |
| DB18030% Acr/Bis (29:1) | MIKX | DB240 |
| 2×PolarSignal™ SYBR Green mix Taq | MIKX | MKG900 |
| Fast site-directed mutagenesis Kit | TIANGEN | KM101 |
| TRIzol | Invitrogen | 15596020 |
| RNAiMAX | Invitrogen | 13778150 |
| HiScript® III RT SuperMix for qPCR (+ gDNA wiper) | Vazyme | R323-01 |
| Passive Lysis Buffer | Promega | E1941 |
| Kinase-Glo® Luminescent Kinase Assay | Promega | V6071 |
| Dual-Luciferase® Reporter Assay System | Promega | E1960 |
| Kinase-Glo® Luminescent Kinase Assay | Promega | V6071 |
| mPER Lysis Buffer | Thermo Fisher | 78501 |
| LumiKine™ Xpress mIFN-β 2.0 | Invivogen | luex-mifnbv2 |
| 2'3'-cGAMP ELISA Kit | Cayman Chemical | 501700 |
| goat serum | Boster Biological | AR1009 |
| Ni-NTA agarose beads | QIAGEN | 30210 |
| PD10 desalting column | GE Healthcare | 52-1308-00 |
| PVDF membranes | Bio-Rad | 1620177 |
| Benzonase | Millipore | E1014 |
| Amicon Ultra 30K | Millipore | C134281 |
| Immobilon Western Chemiluminescent HRP Substrate | Millipore | WBKLS0500 |

**Antibodies**

| **Antibody** | **Source** | **Identifier** |
| --- | --- | --- |
| Anti-Flag-Horseradish peroxidase (HRP) (M2) | Sigma-Aldrich | A8592 |
| Anti-β-actin | Sigma-Aldrich | A1978 |
| Anti-cGAS | Sigma-Aldrich | HPA031700 |
| Anti-hemagglutinin (HA)-HRP | Roche Applied Science | 12013819001 |
| Anti-c-Myc-HRP | Roche Applied Science | 11814150001 |
| Anti-DNA | Millipore | CBL186 |
| Anti-phospho-STAT1 (Tyr701) | Cell Signaling Technology | 9167 |
| Anti-STAT1 | Cell Signaling Technology | 14994 |
| Anti-phospho-TBK1 (Ser172) | Cell Signaling Technology | 5483 |
| Anti-TBK1 | Cell Signaling Technology | 3504 |
| Anti-phospho-IRF3 (Ser396) | Cell Signaling Technology | 4947 |
| Anti-IRF3 | Cell Signaling Technology | 11904 |
| Anti-G3BP1 | Proteintech | 13057-2-AP |
| Anti-TOM20 | Proteintech | 11802-1-AP |
| Anti-PARP | Proteintech | 13371-1-AP |
| Anti-caspase3 | Proteintech | 19677-1-AP |
| Anti-SARS-CoV-2-N protein | Sino Biological | (40588-T62) |
| Goat anti-Rabbit IgG (H+L) cross-adsorbed secondary antibody, HRP | Invitrogen | A-16104 |
| Goat anti-Mouse IgG (H+L) cross-adsorbed secondary antibody, HRP | Invitrogen | A-16072 |
| Goat anti-Mouse IgG (H+L) highly cross-adsorbed secondary antibody, Alexa Fluor 568 | Invitrogen | A-11031 |
| Goat anti-Mouse IgG (H+L) highly cross-adsorbed secondary antibody, Alexa Fluor 488 | Invitrogen | A-11029 |
| Goat anti-Rabbit IgG (H+L) cross-adsorbed secondary antibody, Alexa Fluor 568 | Invitrogen | A-11011 |
| Goat anti-Rabbit IgG (H+L) highly cross-adsorbed secondary antibody, Alexa Fluor 488 | Invitrogen | A-11034 |

**Supplementary Table 2 Sequences of sgRNA, siRNA, dsDNA and qRT-PCR primers.**

**Sequences of sgRNA**

| *cGAS* sgRNA | CACCCGGCCCCCATTCTCGTACGG |
| --- | --- |

**Sequences of siRNA**

| *Scramble* (*SCR*) siRNA | GUUAUCGCAACGUGUCACGUA |
| --- | --- |
| *G3BP1* siRNA #1 | GGGAAUUUGUGAGACAGUA |
| *G3BP1* siRNA #2 | GUCUGAAUGUCGAAGAGAA |

**Sequences of dsDNA**

|  | **Forward** | **Reverse** |
| --- | --- | --- |
| 45 bp ISD | TACAGATCTACTAGTGATCTATGACTGATCTGTACATGATCTACA | TGTAGATCATGTACAGATCAGTCATAGATCACTAGTAGATCTGTA |
| 100 bp  dsDNA | ACATCTAGTACATGTCTAGTCAGTATCTAGTGATTATCTAGACATACATCTAGTACATGTCTAGTCAGTATCTAGTGATTATCTAGACATGGACTCATCC | GGATGAGTCCATGTCTAGATAATCACTAGATACTGACTAGACATGTACTAGATGTATGTCTAGATAATCACTAGATACTGACTAGACATGTACTAGATGT |

**Primers for quantification of RNA**

|  | **Forward** | **Reverse** |
| --- | --- | --- |
| *RPL13A* | GCCATCGTGGCTAAACAGGTA | GTTGGTGTTCATCCGCTTGC |
| *IFNβ* | CAGCAATTTTCAGTGTCAGAAGC | TCATCCTGTCCTTGAGGCAGT |
| *ISG15* | CGCAGATCACCCAGAAGATCG | TTCGTCGCATTTGTCCACCA |
| *IFIT1* | TCAGGTCAAGGATAGTCTGGAG | AGGTTGTGTATTCCCACACTGTA |
| *IFIT2* | GGAGGGAGAAAACTCCTTGGA | GGCCAGTAGGTTGCACATTGT |
| *mCherry* | CACGAGTTCGAGATCGAGGG | CAAGTAGTCGGGGATGTCGG |
| SARS-CoV-2  *N protein* | GGGGAACTTCTCCTGCTAGAAT | CAGACATTTTGCTCTCAAGCTG |
| SARS-CoV-2  *ORF1ab* | CCCTGTGGGTTTTACACTTAA | ACGATTGTGCATCAGCTGA |

**Primers for quantification of cytosolic DNA or cGAS-bound DNA**

|  | **Forward** | **Reverse** |
| --- | --- | --- |
| *mtDNA1* | CACCCAAGAACAGGGTTTGT | TGGCCATGGGTATGTTGTTAA |
| *mtDNA2* | CTATCACCCTATTAACCACTCA | TTCGCCTGTAATATTGAACGTA |
| *mtDNA3* | AATCGAGTAGTACTCCCGATTG | TTCTAGGACGATGGGCATGAAA |
| *mtDNA4* | AATCCAAGCCTACGTTTTCACA | AGTATGAGGAGCGTTATGGAGT |
| *mCherry* | CACGAGTTCGAGATCGAGGG | CAAGTAGTCGGGGATGTCGG |

**Supplementary Table 3 Reagent formulations.**

| **Reagent** | **Formulation** |
| --- | --- |
| Lysis buffer for recombinant protein purification | 50 mM Tris-HCl pH 7.5, 500 mM NaCl, 20 mM imidazole, 0.035% β-ME, 5% Glycerol and protease inhibitors |
| Wash buffer for recombinant protein purification | 50 mM Tris-HCl pH 7.5, 500 mM NaCl, 40 mM imidazole, 0.035% β-ME, 5% Glycerol and protease inhibitors |
| Elution buffer for recombinant protein purification | 50 mM Tris-HCl pH 7.5, 500 mM NaCl, 250 mM imidazole, 0.035% β-ME, 5% Glycerol and protease inhibitors |
| Storage buffer for recombinant protein purification | 20 mM Tris-HCl pH 7.5, 300 mM NaCl and 1M DTT |
| Extraction buffer for cytosolic DNA | 150 mM NaCl, 50 mM HEPES pH 7.4, 25 μg/ml digitonin (MCE) |
| Low salt lysis buffer (LSB) | 50 mM HEPES, 150 mM NaCl, 1 mM EDTA, 10% glycerol, 1.5 mM MgCl_2_, and 1% Triton X-100 |
| Elution buffer for cGAS-bound DNA | 50 mM Tris, pH 8.0, 1 mM EDTA, 1% SDS, 50 mM NaHCO_3_ |
| LLPS buffer | 5 mM Tris-HCl pH 7.5, 200 mM NaCl, 1 mM DTT |
