# Supplementary material for: Phase-separated nucleocapsid protein of SARS-CoV-2 suppresses cGAS-DNA recognition by disrupting cGAS-G3BP1 complex
Source: Signal Transduct Target Ther. 2023 Apr 26;8:170. doi: 10.1038/s41392-023-01420-9 (PMC10131525; doi:10.1038/s41392-023-01420-9)

Supplementary Materials for

**Phase-separated nucleocapsid protein of SARS-CoV-2 suppresses cGAS-DNA recognition by disrupting cGAS-G3BP1 complex**

Sihui Cai, Chenqiu Zhang, Zhen Zhuang, Shengnan Zhang, Ling Ma, Shuai Yang, Tao Zhou, Zheyu Wang, Weihong Xie, Shouheng Jin, Jincun Zhao, Xiangdong Guan, Jianfeng Wu, Jun Cui, Yaoxing Wu

Correspondence to: Yaoxing Wu (wuyaox5@mail.sysu.edu.cn), Xiangdong Guan (guanxd@mail.sysu.edu.cn), Jianfeng Wu (wujianf@mail.sysu.edu.cn) or Jun Cui (cuij5@mail.sysu.edu.cn)

**This PDF file includes:**

Original and uncropped films of Western blots


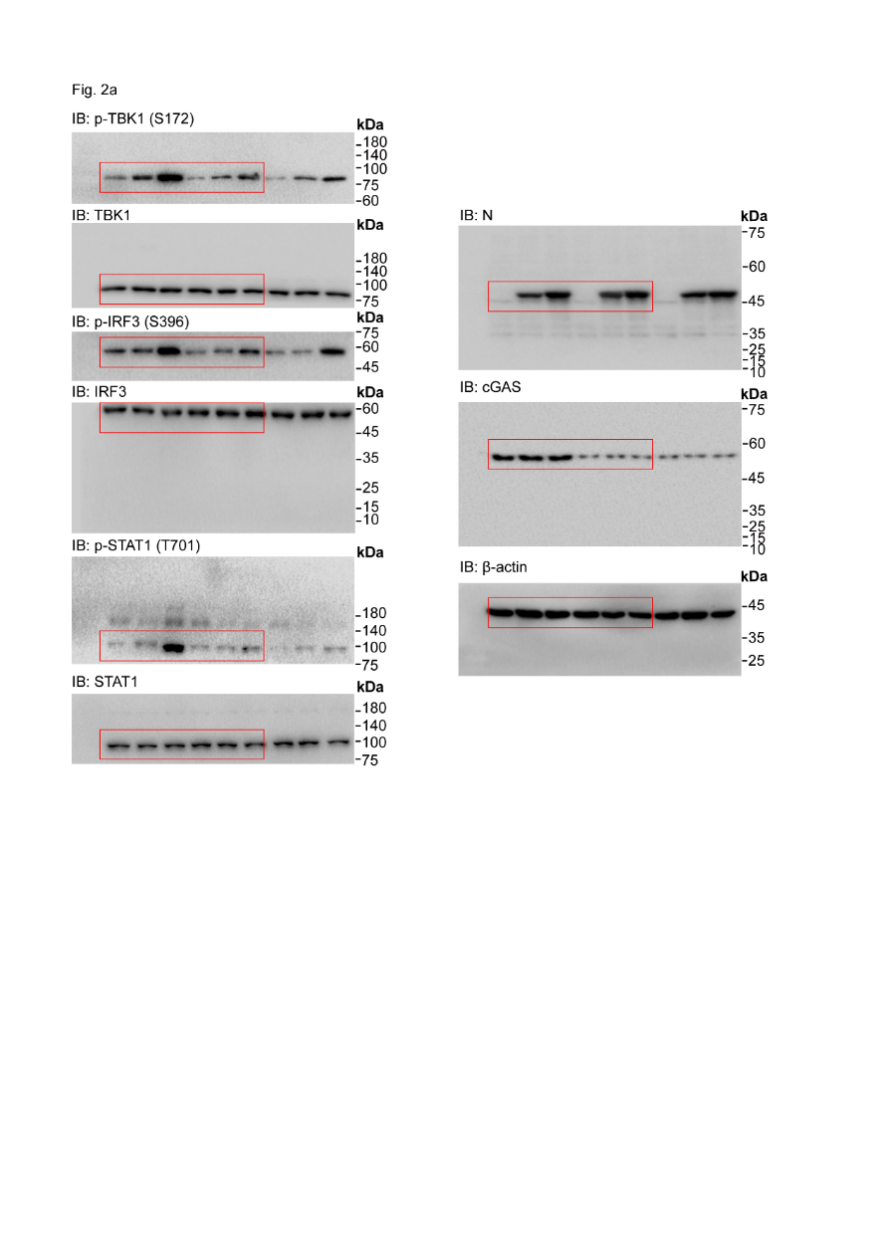


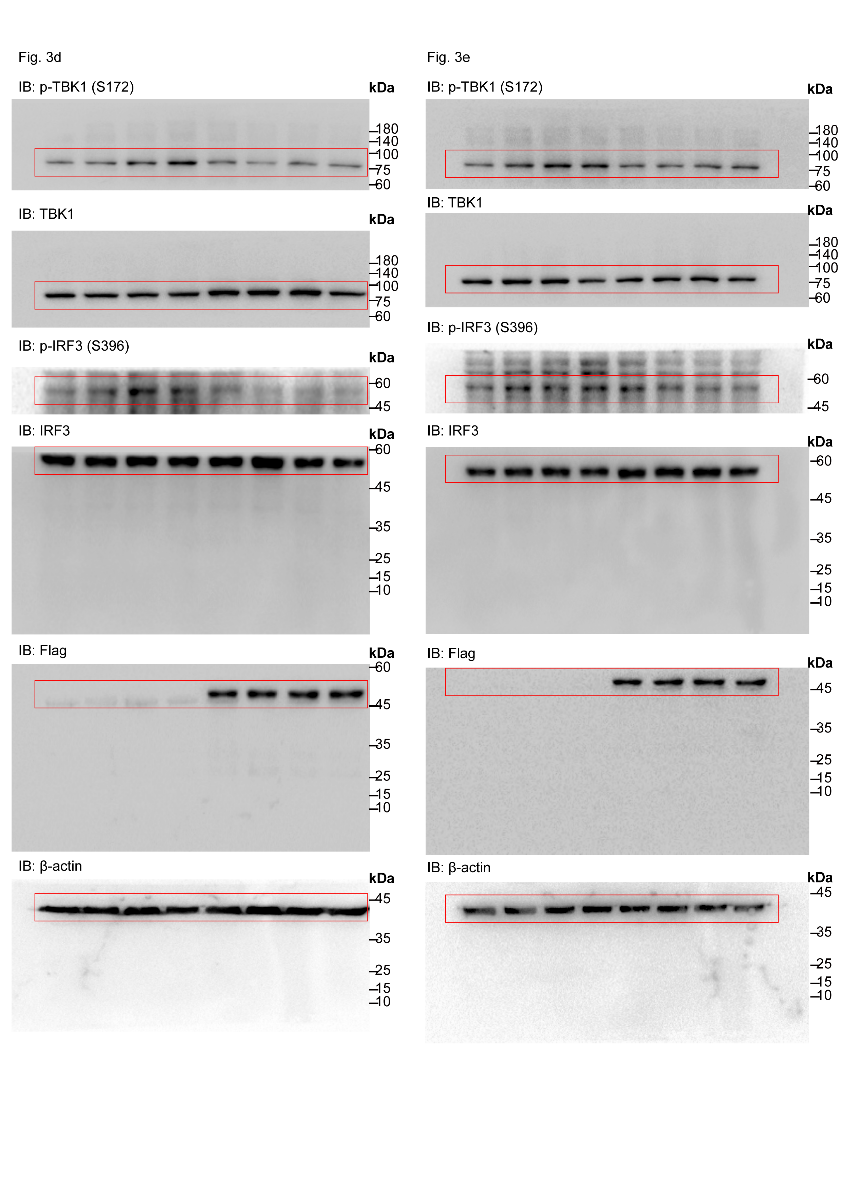

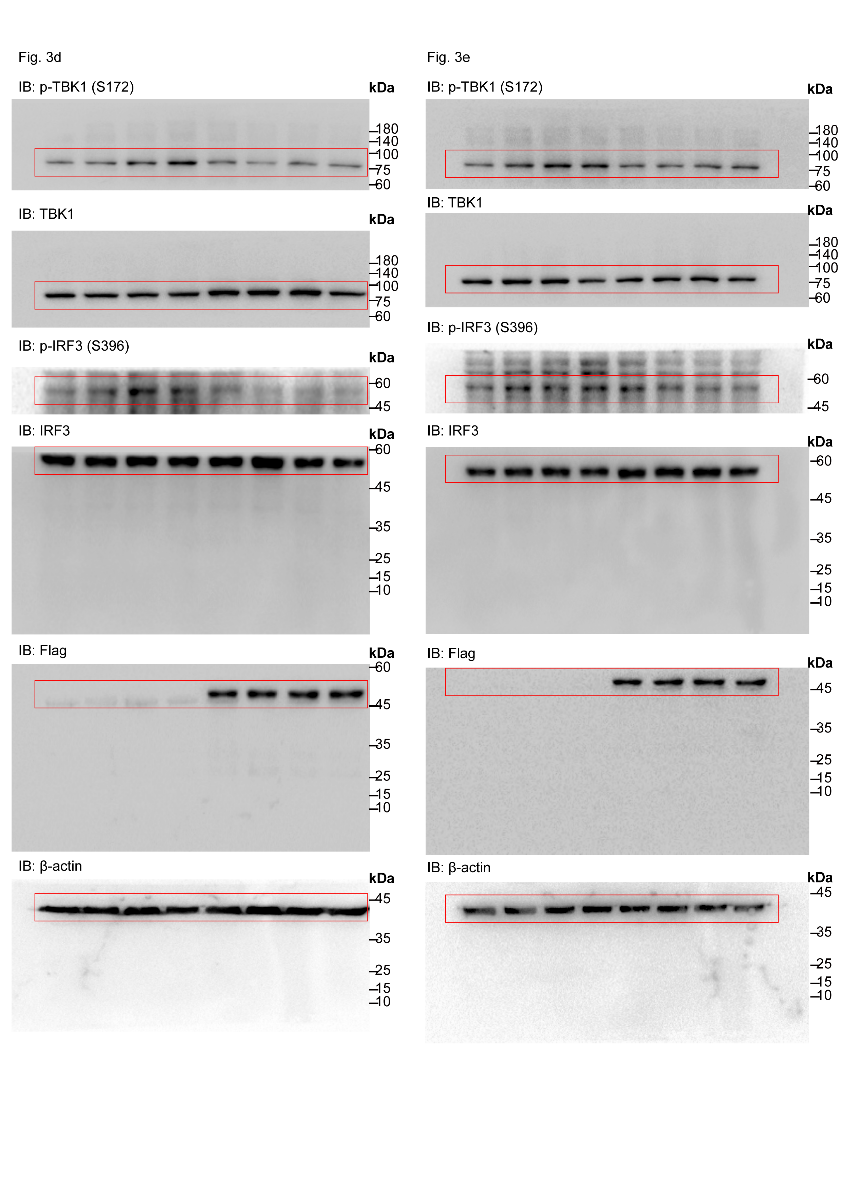
**
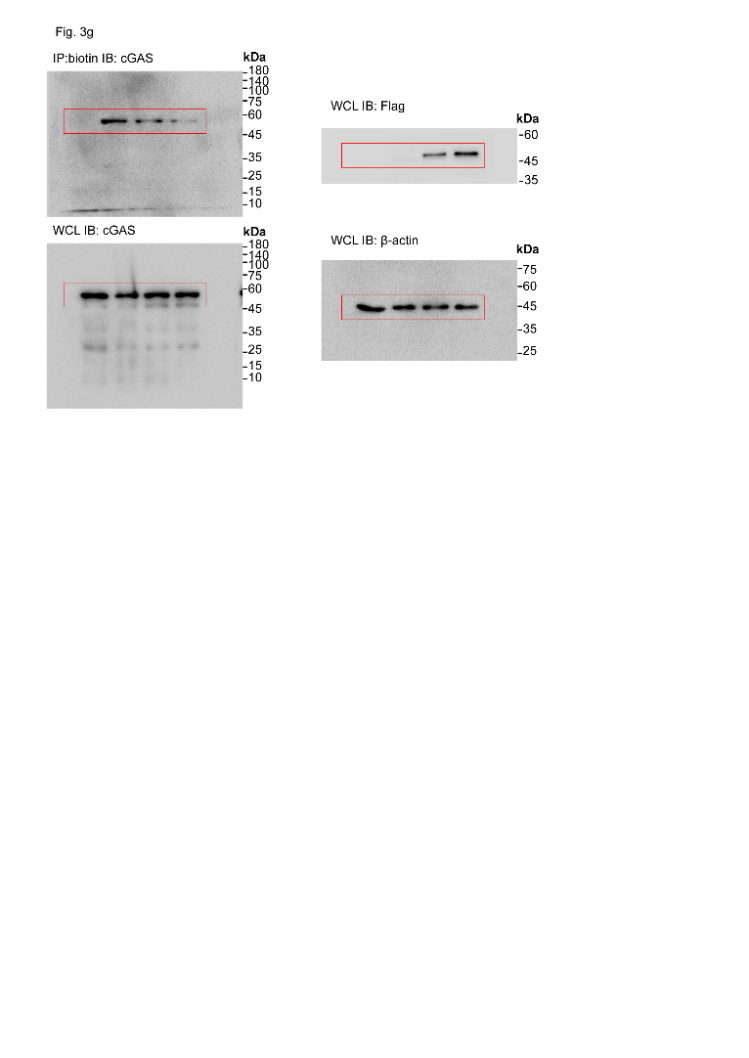
**


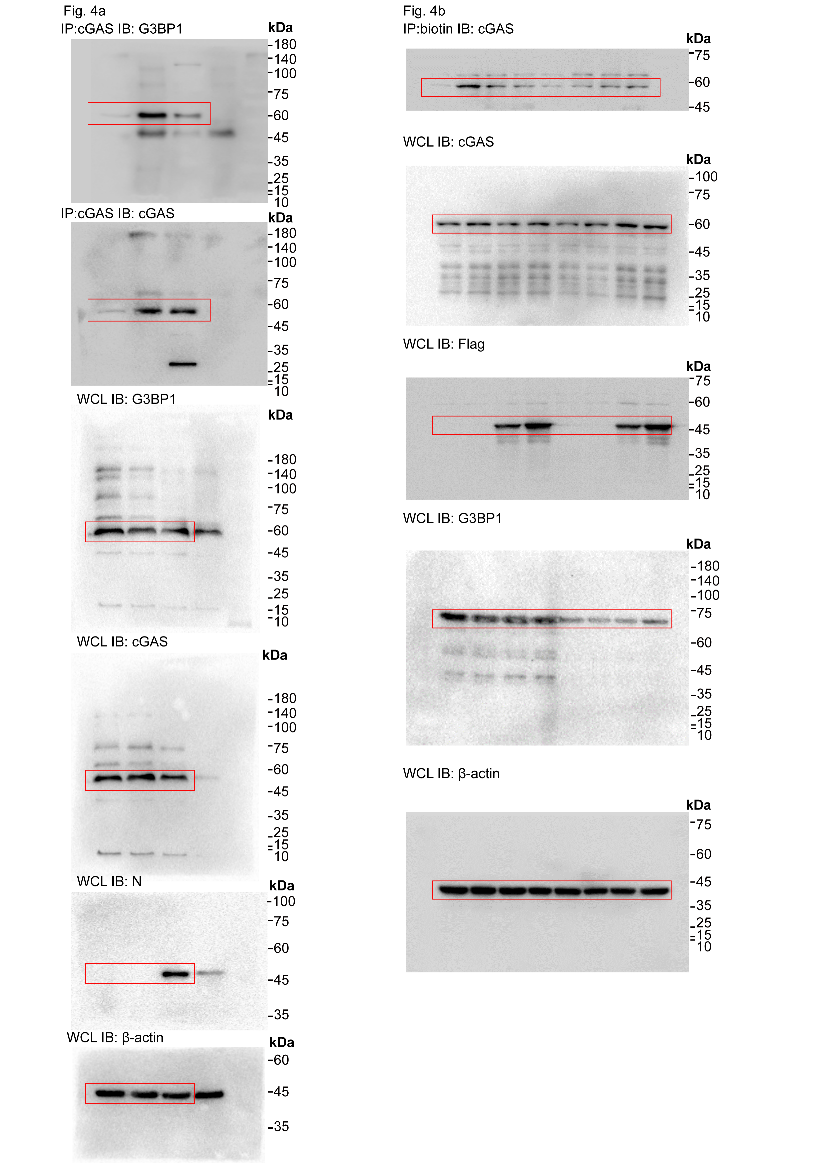

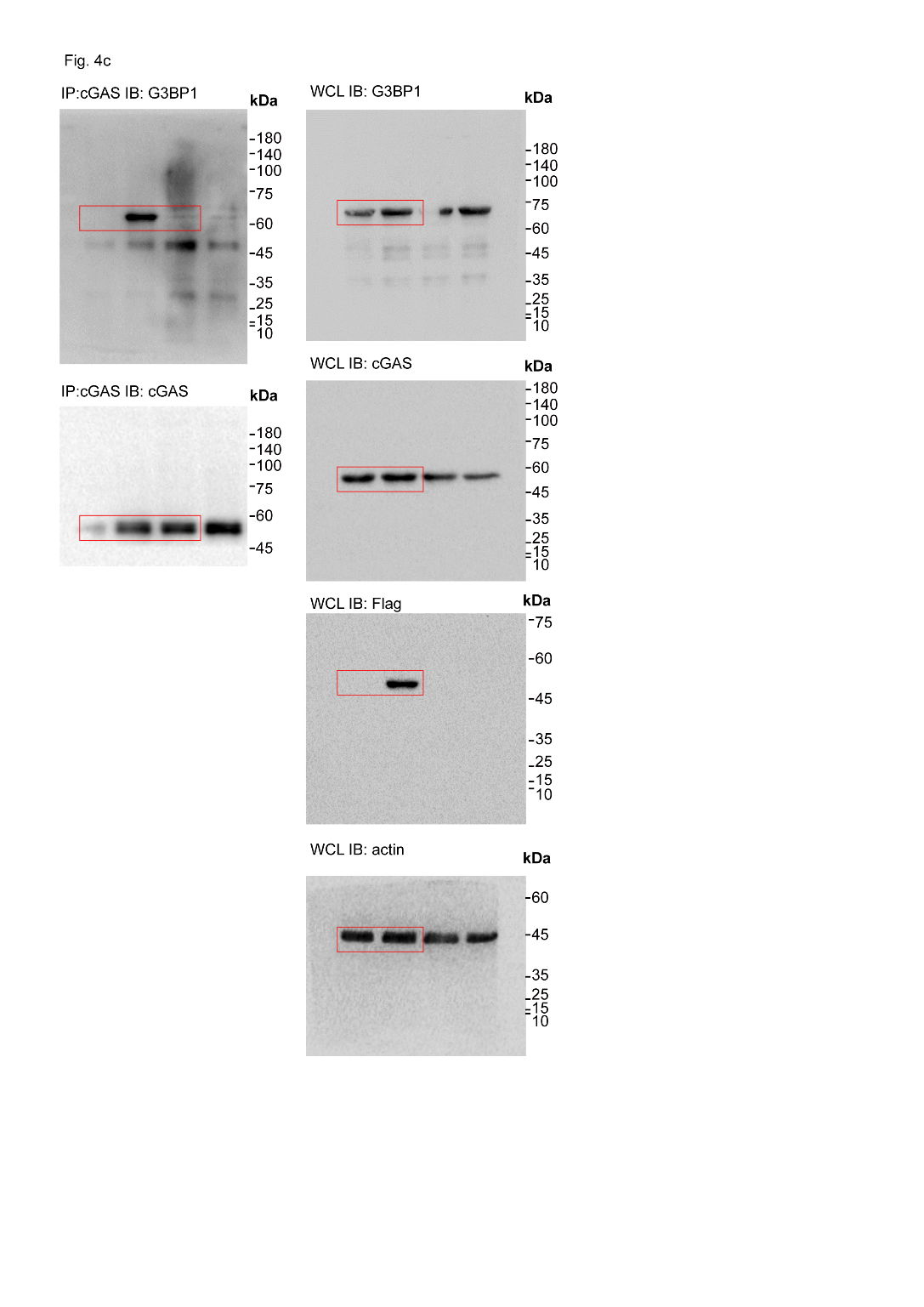

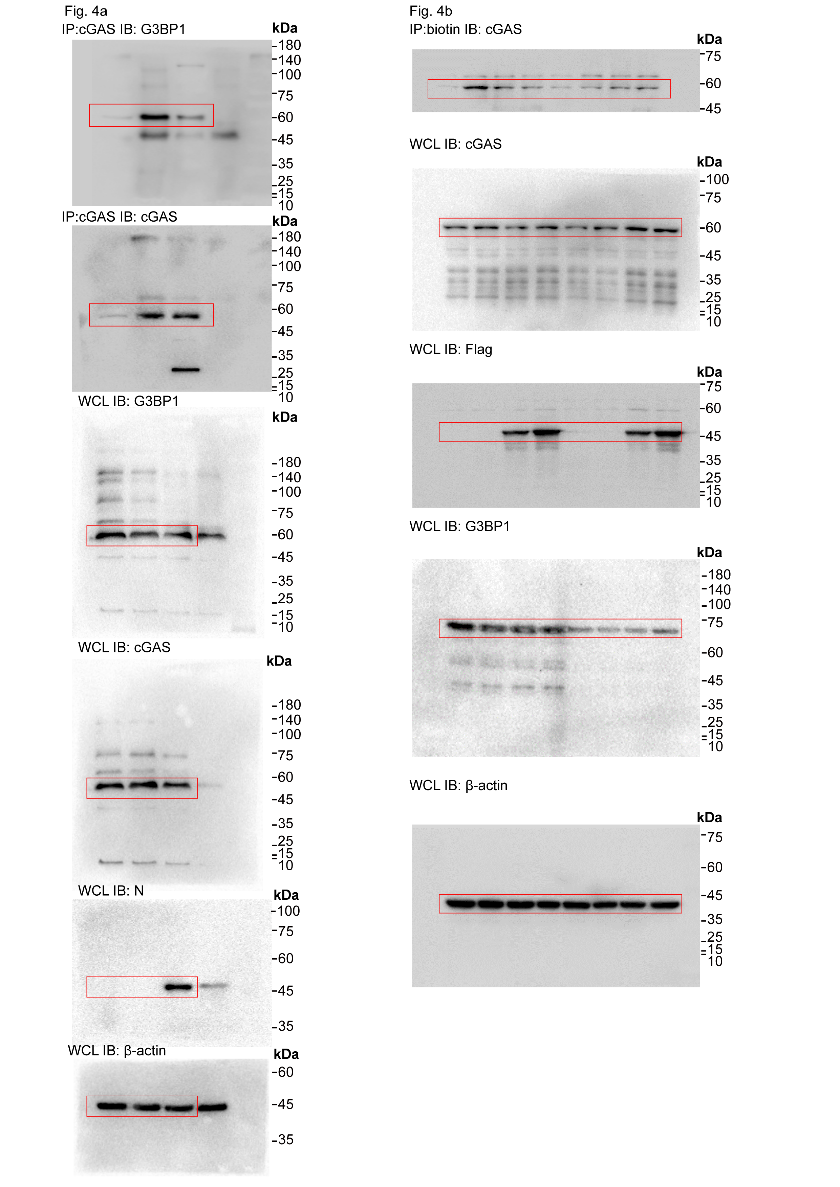


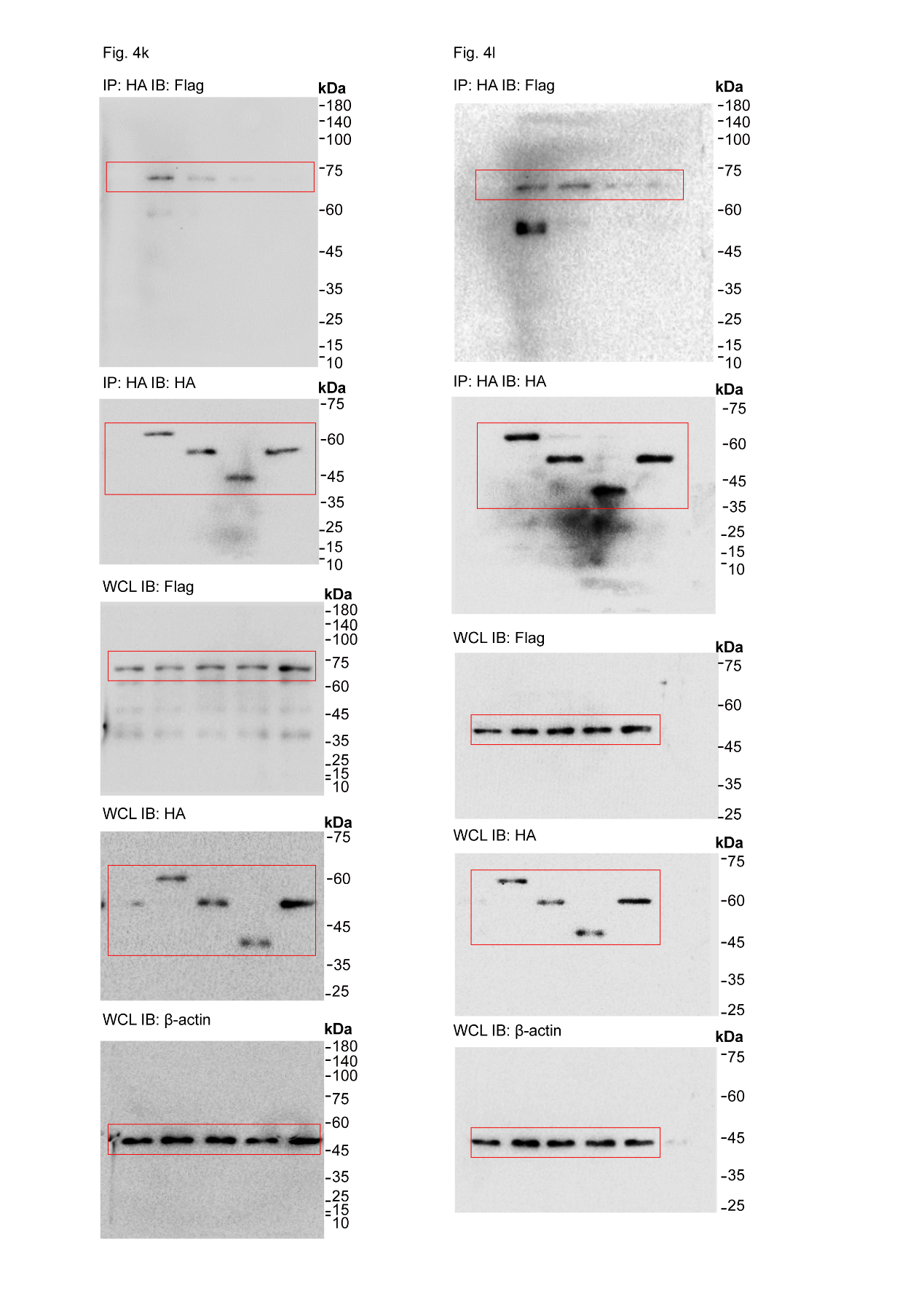

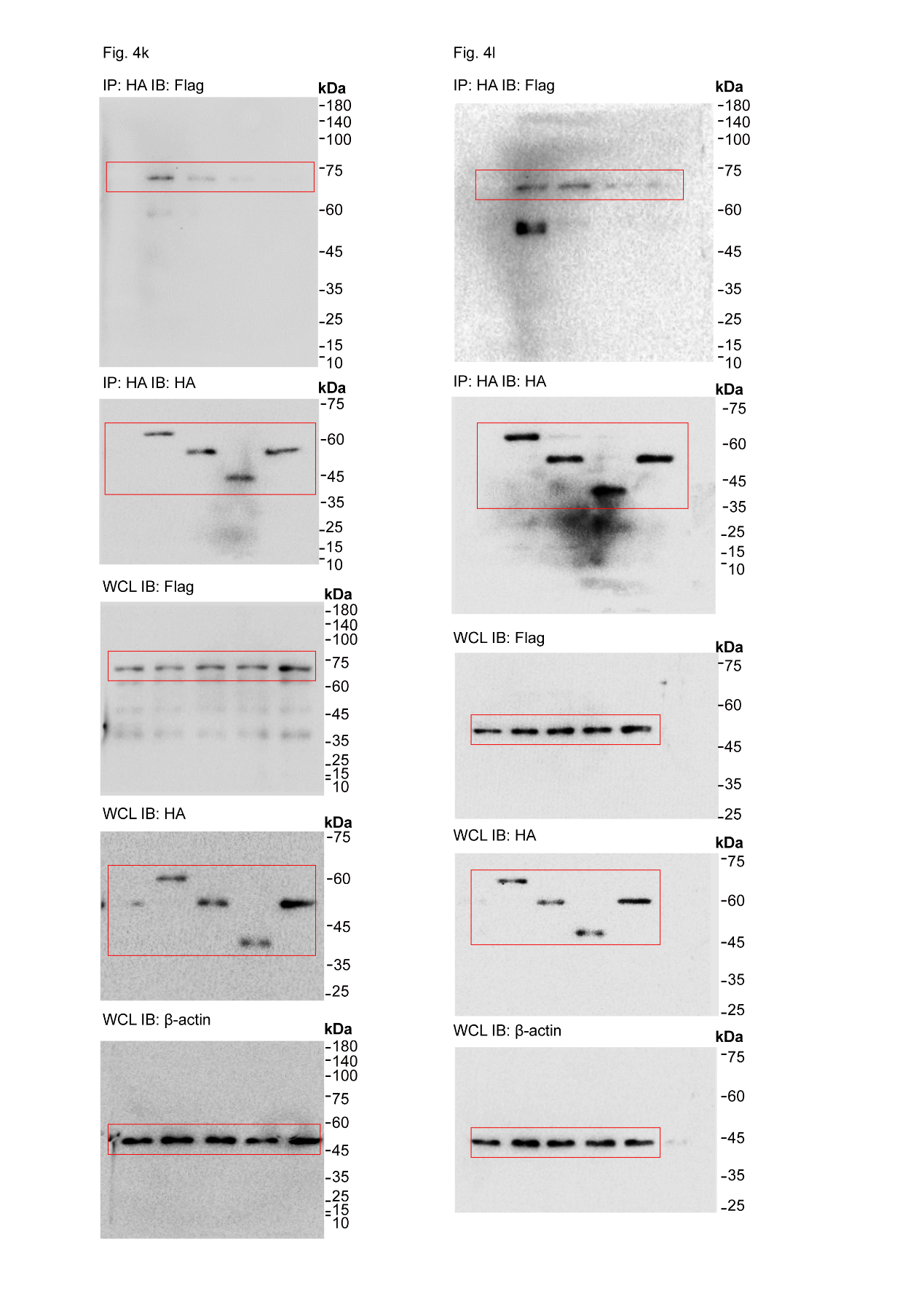


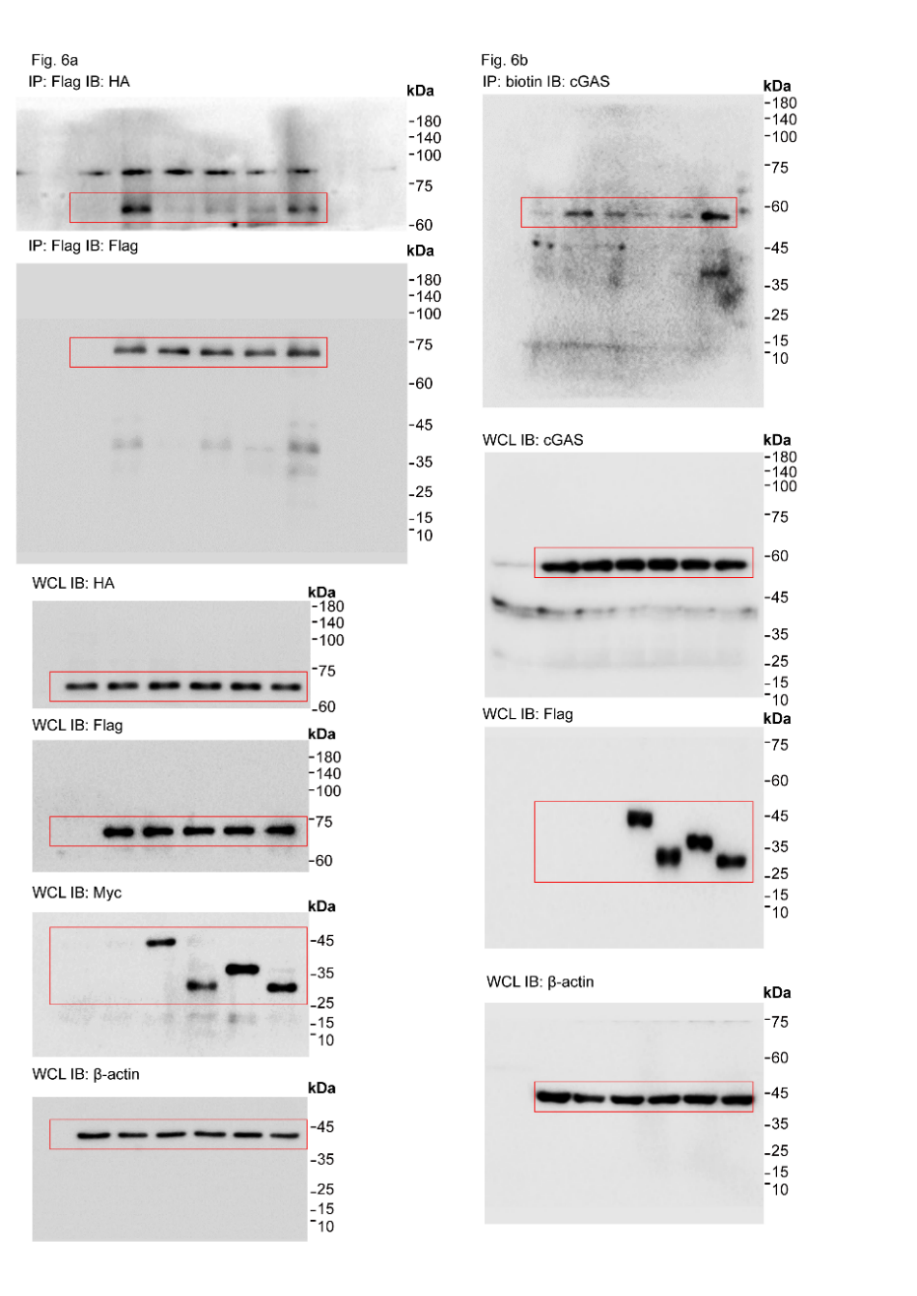

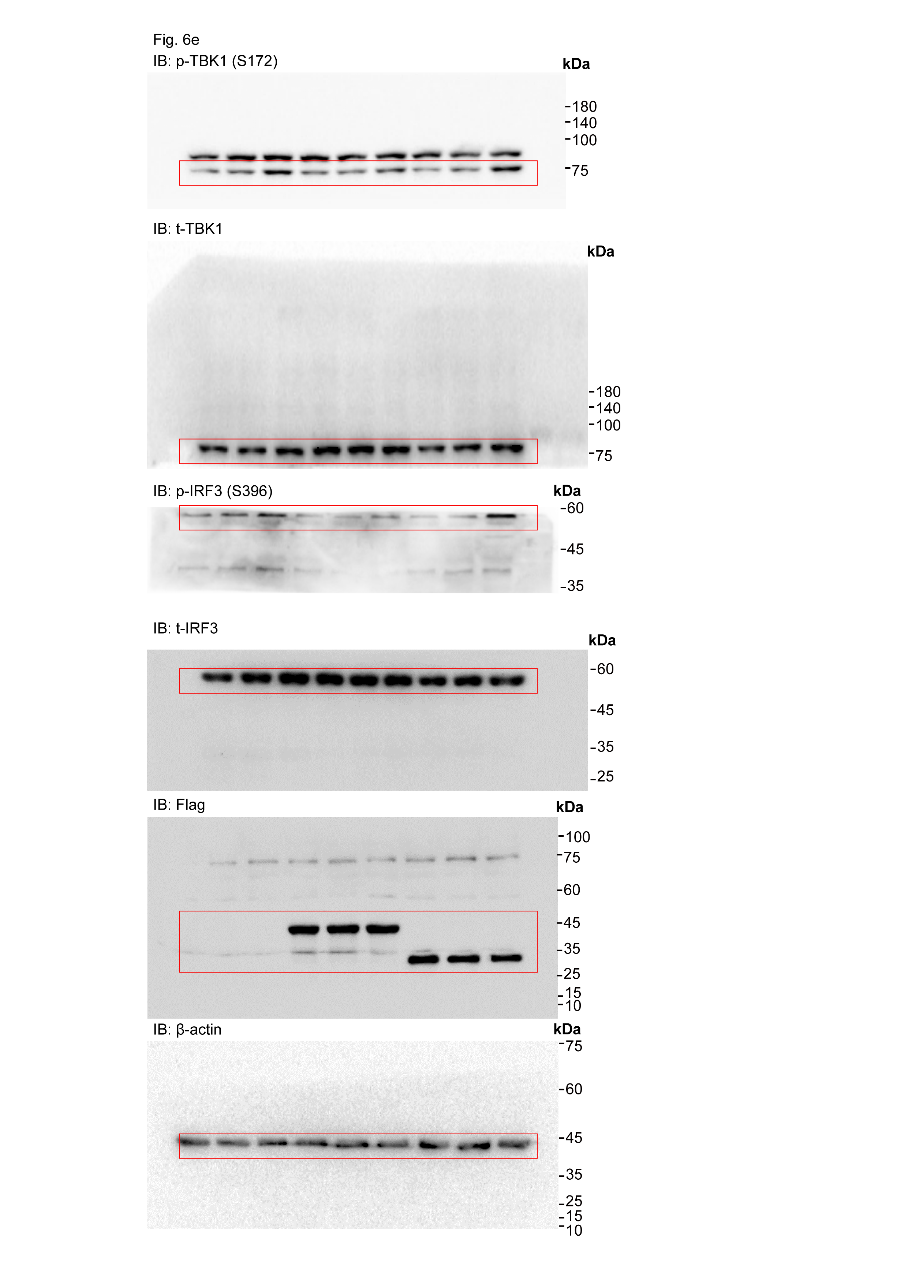

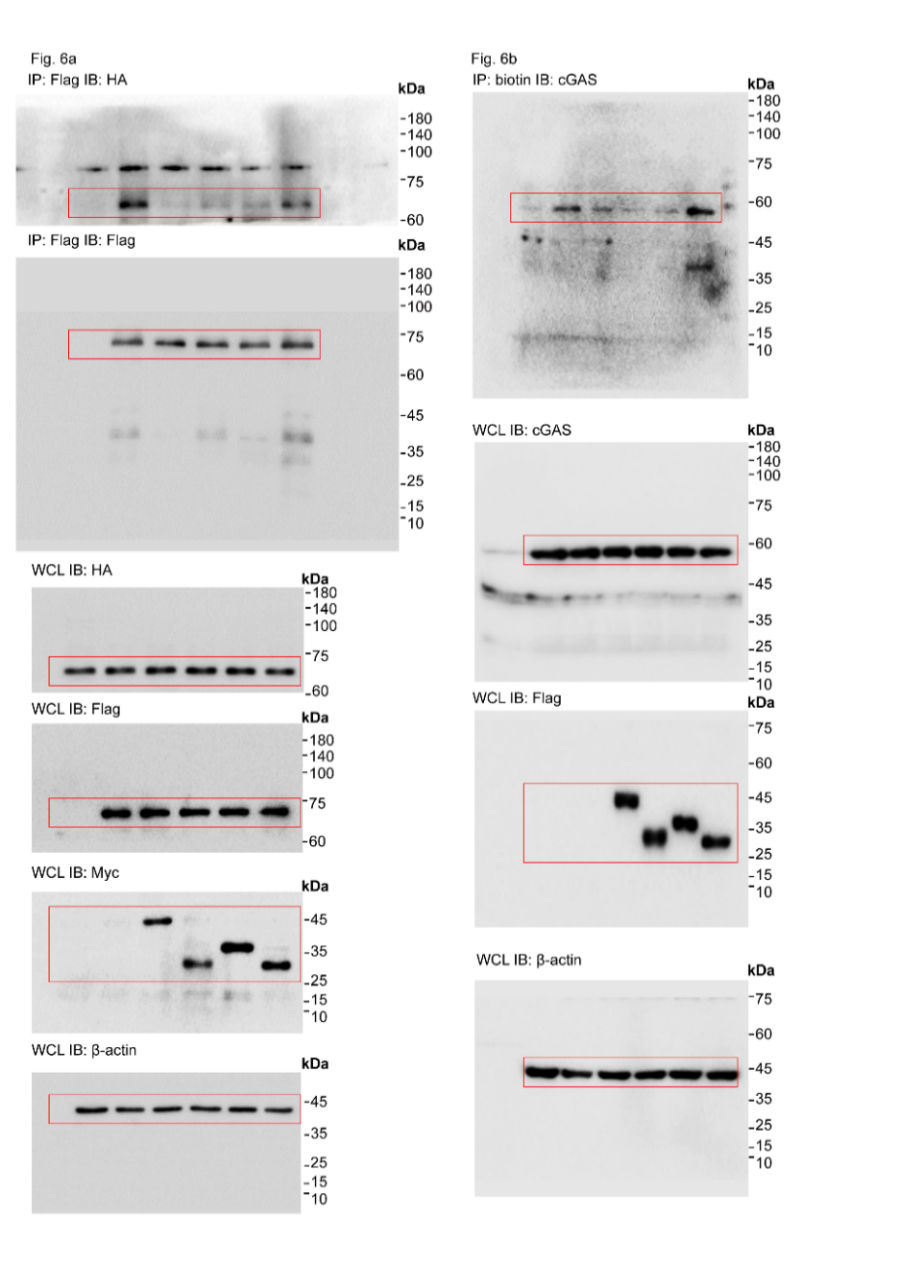


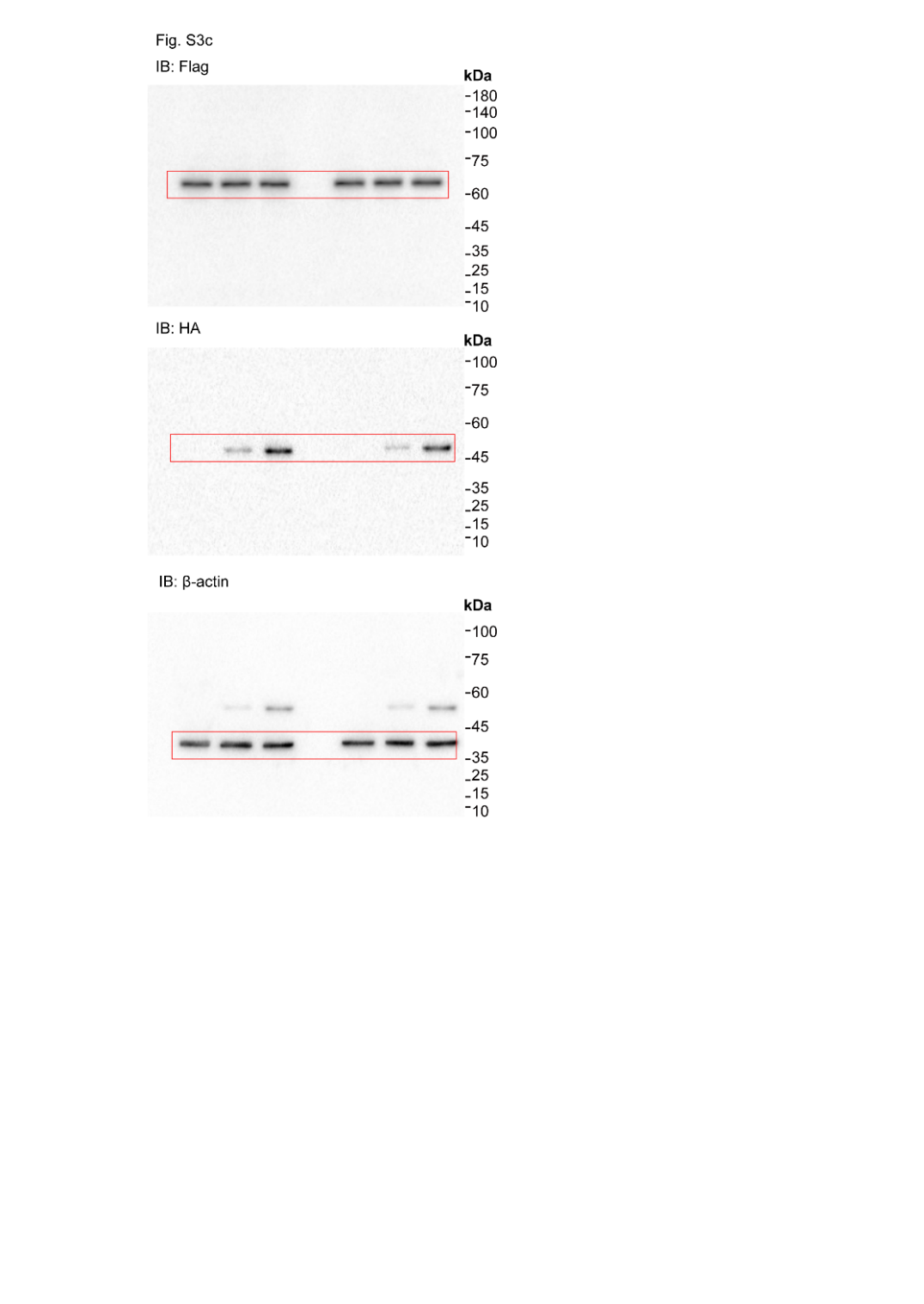

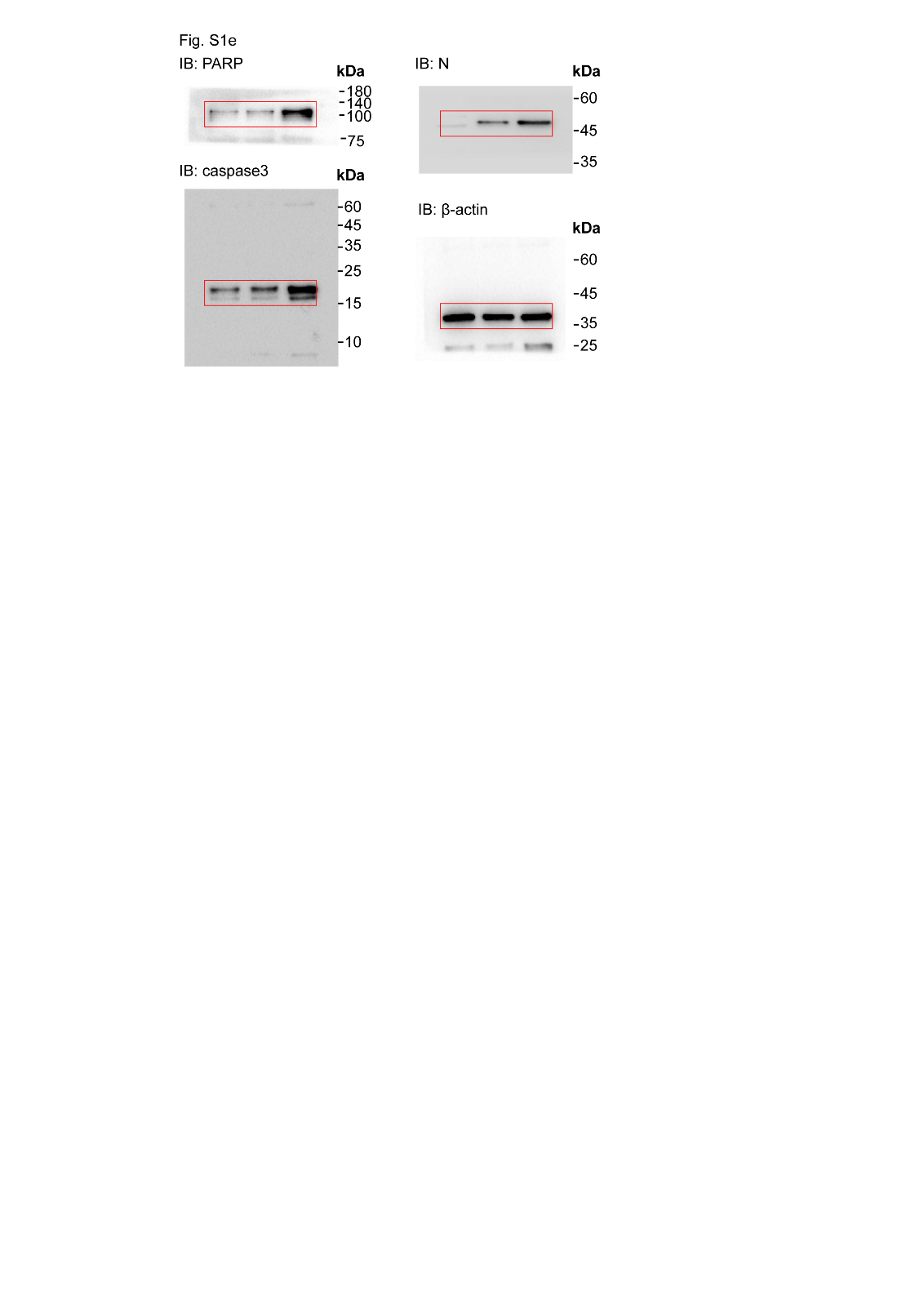


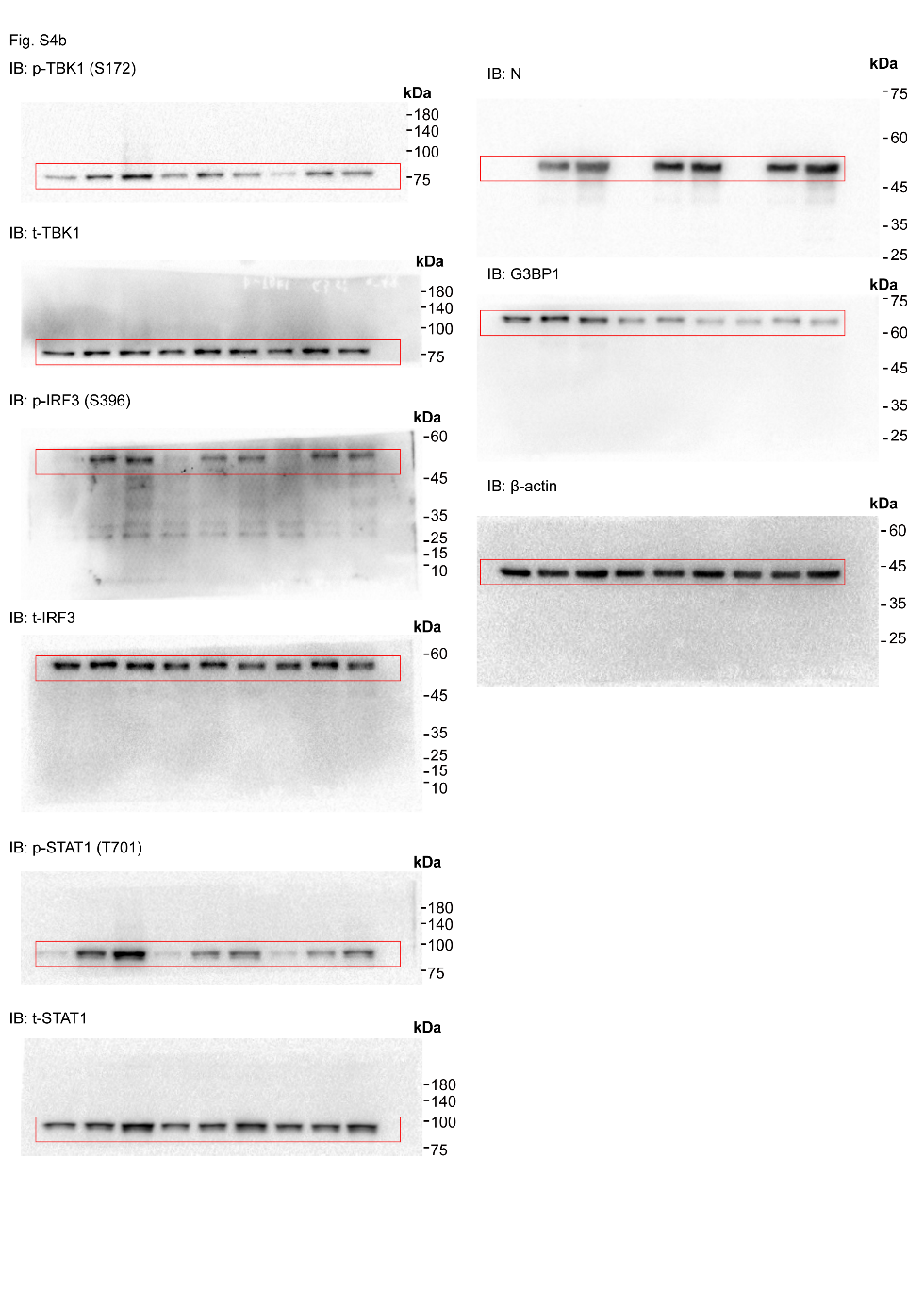


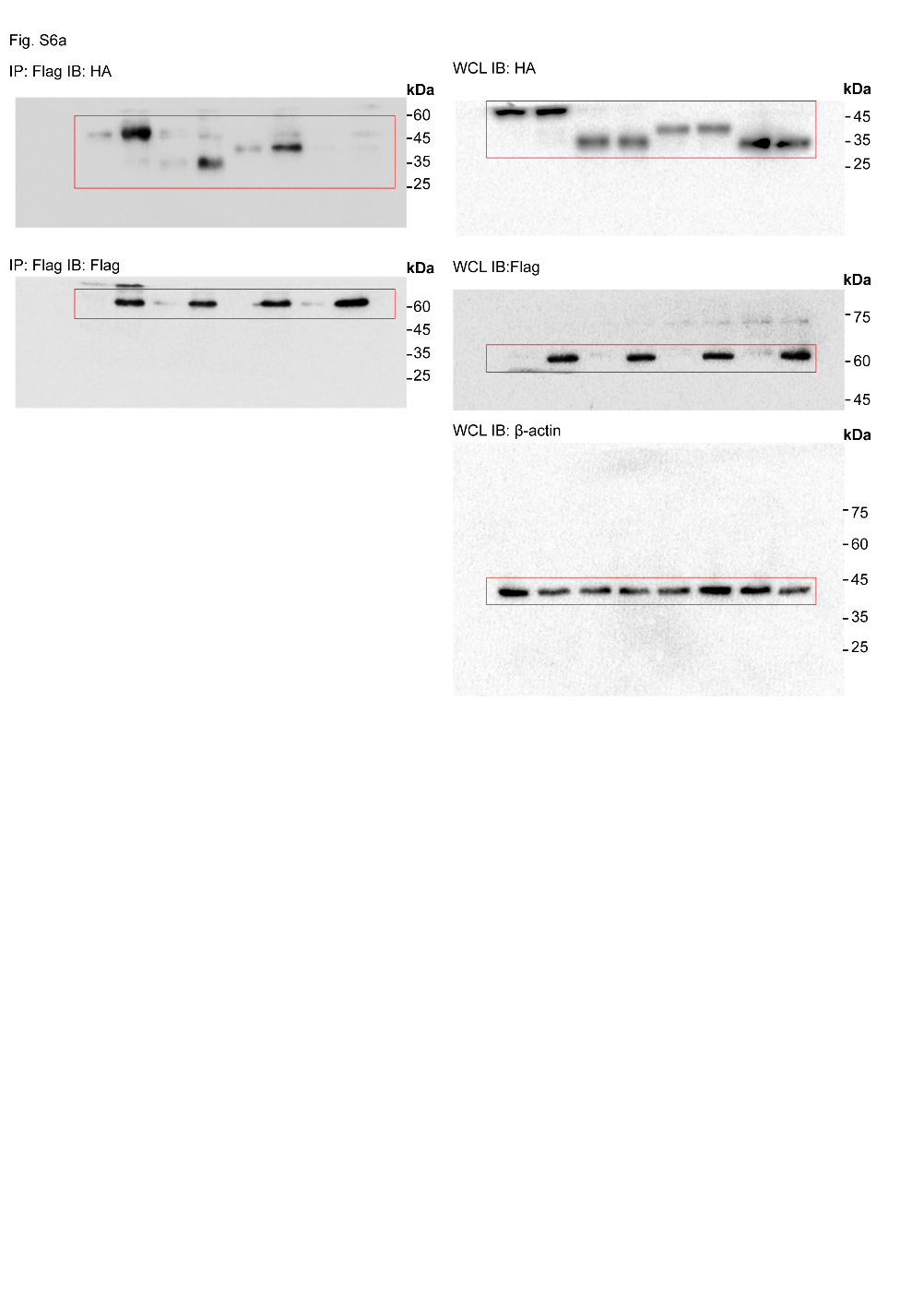

Supplement: Supplementary file 2 — Supplementary Materials for uncropped figure [file 41392_2023_1420_MOESM2_ESM.docx]
